# Peroxiredoxin 1 regulates crosstalk between pyroptosis and autophagy in oral squamous cell carcinoma leading to a potential pro-survival

**DOI:** 10.1038/s41420-023-01720-7

**Published:** 2023-11-25

**Authors:** Meilin Ye, Ting Liu, Shanshan Liu, Rong Tang, Hongrui Liu, Fan Zhang, Shenglei Luo, Minqi Li

**Affiliations:** 1 Department of Bone Metabolism, School and Hospital of Stomatology, Cheeloo College of Medicine, Shandong University & Shandong Key Laboratory of Oral Tissue Regeneration & Shandong Engineering Research Center of Dental Materials and Oral Tissue Regeneration & Shandong Provincial Clinical Research Center for Oral Diseases, Jinan, China; 2 Center of Osteoporosis and Bone Mineral Research, Shandong University, Jinan, China; 3 Department of Orthodontics, School and Hospital of Stomatology, Cheeloo College of Medicine, Shandong University & Shandong Key Laboratory of Oral Tissue Regeneration & Shandong Engineering Research Center of Dental Materials and Oral Tissue Regeneration & Shandong Provincial Clinical Research Center for Oral Diseases, Jinan, China; 4 Department of Oral and Maxillofacial Surgery, The Second Hospital, Cheeloo College of Medicine, Shandong University, Jinan, China

**Keywords:** Oral cancer detection, Oral cancer detection

## Abstract

Peroxiredoxin 1 (Prdx1), a vital antioxidant enzyme, has been proven to play an important role in the occurrence and development of cancers, but its effects on oral squamous cell carcinoma (OSCC) remain unclear. Here, we performed bioinformatics analysis and immunohistochemical (IHC) staining to confirm that Prdx1 was higher in OSCC tissues than in normal tissues. Consistently, RT-PCR and Western blot showed elevated Prdx1 expression in OSCC cell lines compared to human oral keratinocytes (HOK), which could be knockdown by small interfering RNA (siRNA) and Lentiviral vector delivery of short hairpin RNA (shRNA). Prdx1 silencing significantly blocked OSCC cell proliferation and metastasis, as evidenced by the CCK8, colony formation, in vivo tumorigenesis experiment, wound healing, transwell assays, and changes in migration-related factors. siPrdx1 transfection increased intracellular reactive oxygen species (ROS) levels and provoked pyroptosis, proved by the upregulation of pyroptotic factors and LDH release. Prdx1 silencing ROS-independently blocked autophagy. Mature autophagosome failed to form in the siPrdx1 group. Up-regulated autophagy limited pyroptosis triggered by Prdx1 deficiency, and down-regulated pyroptosis partly reversed siPrdx1-induced autophagy defect. Collectively, Prdx1 regulated pyroptosis in a ROS-dependent way and modulated autophagy in a ROS-independent way, involving the crosstalk between pyroptosis and autophagy.

## Introduction

Oral cancer ranks among the top eight malignant tumors globally, with over 377,000 new cases reported in 2020, of which more than 90% are oral squamous cell carcinoma (OSCC) [1]. OSCC is marked by poor prognosis [2]. Specific oncogene alterations may enhance the invasive and metastatic capabilities of OSCC cells, contributing to the high incidence of local invasive growth and cervical lymph node metastasis in OSCC patients [3]. Consequently, identifying key endogenous aberrations involved in OSCC pathogenesis is crucial for discovering new biomarkers or developing novel targeted therapies.

Reactive oxygen species (ROS) are by-products of oxygen consumption and cellular metabolism, functioning as bioactive mediators to drive cellular activities and maintained in a dynamic balance by various reduction–oxidation systems at physiological concentrations. On the other hand, excessive ROS can damage biological components, thus, antioxidant enzymes are compensatorily upregulated to protect cells from ROS toxicity [4]. Numerous antioxidant enzyme systems were identified as promising anti-tumor targets [4], for example, the inhibitors of the thioredoxin (Trx) system have been advanced into clinical trials for cancer treatment [5]. The peroxiredoxin (Prdx) family, another vital oxidative defense system, that serves as the direct substrate of Trx, remains lacking in research. Prdx1 is the most studied among the Prdx family members, whose abnormal expression has been observed in various tumors and precancerous lesions, including OSCC [6, 7], esophageal cancer [8], and others [8]. Cancer patients with stronger Prdx1 expression often suffer from worse outcomes [9, 10]. In a study involving 68 OSCC patients, Prdx1 expression correlated with clinical stage, lymphatic metastasis, and pathological grade, indicating its potential as a prognostic biomarker for survival [7]. Although Prdx1 has been implicated in cancer cell proliferation, metastasis, invasion, angiogenesis, and other processes [8, 11, 12], the underlying molecular mechanisms remain incompletely understood.

Apoptosis-targeted drugs often struggle to achieve efficacy in OSCC due to safety requirements and the induction of tolerance. Pyroptosis, a novel type of programmed cell death (PCD) characterized by inflammatory features, such as proinflammatory cytokine activation, membrane pore formation, fluid influx, cell swelling, and lysis, has emerged as a potential target for drug discovery [13]. In canonical pyroptosis, NLRP3, activated by pathogenic stimuli, recruits and matures pro-caspase-1 to form inflammasomes, which then releases N-terminal fragments from gasdermin D (GSDMD) to form pores in the cell membrane. Interleukin (IL)-1β and IL-18 are also cleaved by caspase-1 and leaks through the cellular pores. Considering the roles of the Prdx family in inflammation [14], and the inflammatory characteristic of pyroptosis, we wonder whether a link exists between Prdx1 and pyroptosis.

Autophagy, another type of PCD, plays a crucial role in meeting metabolic needs and maintaining cellular homeostasis [15, 16]. This lysosome-dependent degrative process can cut down damaged organelles caused by stimuli such as ROS, thus enhancing cellular resistance to harmful environments. The mechanistic target of rapamycin kinase (mTOR) (288kD), an upstream suppressor, functions in its phosphorylated form (p-mTOR) (289kD), while Beclin1 and p62 facilitate autophagosome formation. Light chain 3B (LC3B) embeds into the autophagosome membrane and dissolves enzymatically, with the conversion from LC3B-I (19kD) to LC3B-II (17kD) representing autophagosome maturation [17]. In hepatocellular carcinoma, silencing Prdx1 expression has been shown to inhibit autophagy [18]. In colorectal cancer, Prdx2 deficiency exhibited antitumor effects and suppressed autophagy [19]. We want to fill in the blank on the regulation of Prdx1 on autophagy in OSCC.

In our study, we aim to elucidate the influence of Prdx1 on OSCC cell behavior and investigate the regulatory mechanisms of Prdx1 in OSCC cells from the perspectives of autophagy and pyroptosis. This research may contribute to our understanding of Prdx1 and identify a potential candidate for OSCC therapy.

## Results

### Prdx1 is highly expressed in human OSCC tissues and cells

**Fig. 1:**
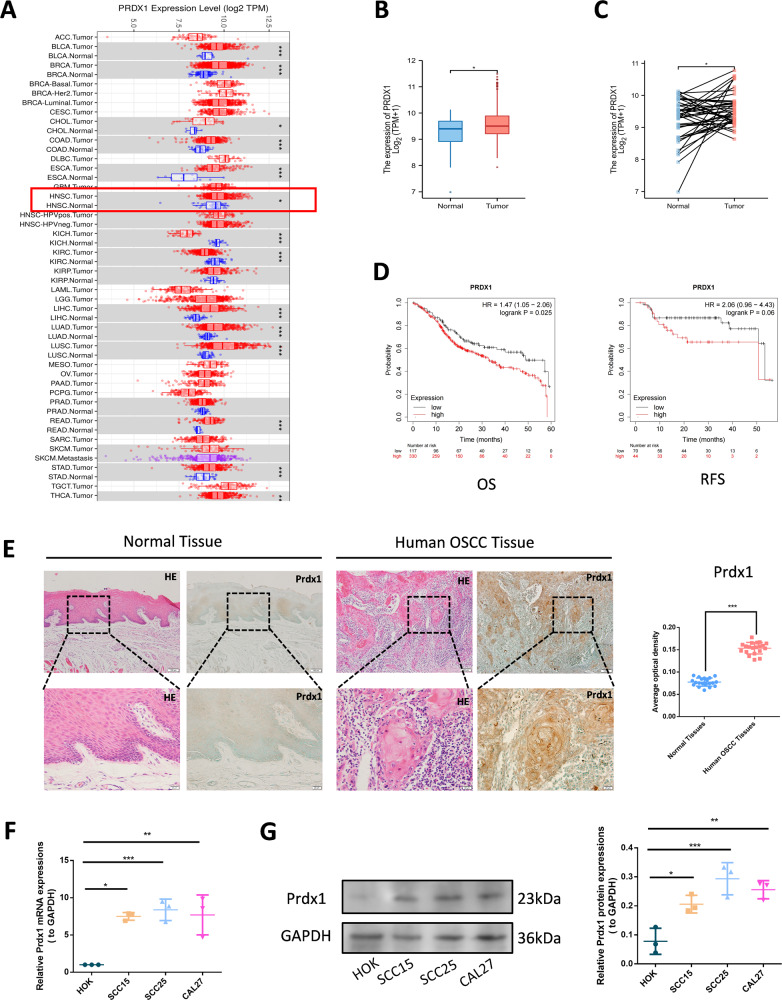
Prdx1 is up-regulated in OSCC tissues and cell lines. **A**–**C** Differentially expressed gene analysis of Prdx1 expression in OSCC tissue and adjacent normal tissue. **D** Association between Prdx1 mRNA expression and prognosis in HNSC. **E** HE staining and IHC staining of Prdx1 in human OSCC tissues and adjacent non-tumor tissues as normal tissues. **F** RT-PCR analysis of Prdx1 expression in HOK cell and OSCC cells. **G** Western blot analysis of Prdx1 expression in HOK cells and OSCC cells. The scale bar was 20 or 100 µm. The results are shown as the mean ± SD (*n* ≥ 3), ns, non-significant difference; *p < 0.05; **p < 0.01; ***p < 0.001.

Analysis using the tumor immune estimation resource (TIMER) demonstrated variable expression of Prdx1 in pan-cancer, with notable overexpression in head and neck squamous cell carcinoma (HNSC) (Fig. 1A). Both unpaired and paired analyses of data from The Cancer Genome Atlas (TCGA) database indicated upregulation of Prdx1 in OSCC cohorts (Fig. 1B, C). HNSC patients were stratified into two groups based on high or low Prdx1 expression. Kaplan–Meier survival curves revealed that patients with elevated Prdx1 mRNA levels were associated with decreased OS (log-rank *P* = 0.025, HR = 1.47, 95% CI = 1.05–2.06) and recurrence-free survival (RFS) (log-rank *P* = 0.06, HR = 2.06, 95% CI = 0.96–4.43) (Fig. 1D). IHC analysis further confirmed significantly higher Prdx1 expression in human OSCC tissues compared to adjacent non-tumor tissues (Fig. 1E). Additionally, increased Prdx1 expression in OSCC cell lines (SCC15, SCC25, and CAL27) relative to the human oral keratinocyte cell line (HOK) was validated by western blot and RT-PCR (Fig. 1F, G).

### Prdx1 knockdown inhibits the proliferation and migration of OSCC cells

**Fig. 2:**
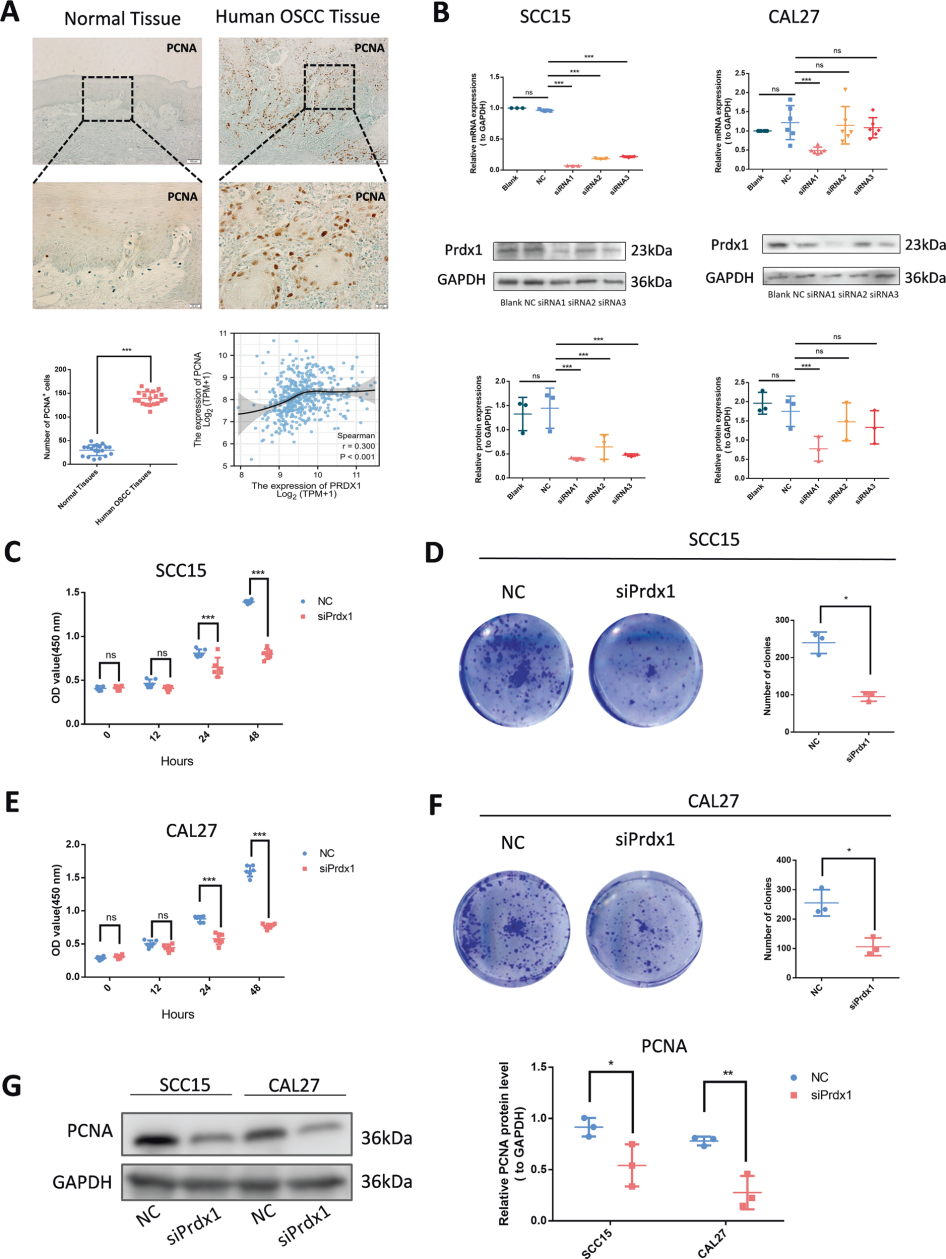
Prdx1 knockdown inhibits the proliferation of OSCC cells. **A** IHC staining of PCNA in human OSCC tissues and normal tissues. The scale bar was 20 or 100 µm. **B** RT-PCR and western blot analysis of the levels of Prdx1 in NC groups and siPrdx1 groups in SCC15 and CAL27. **C**, **E** Cell viability was detected by CCK8 for 12, 24, and 48 h after transfection in SCC15 and CAL27 cells. **D**, **F** Colone formation of NC and siPrdx1 groups for 14 days. **G** Western blot analysis of PCNA expression in OSCC cells. The results are shown as the mean ± SD (*n* = 3), ns non-significant difference, *p < 0.05; **p < 0.01; ***p < 0.001.

Continuous section staining revealed an increase in PCNA levels in human OSCC tissues with high Prdx1 expression and bioinformatic analysis suggested a potential correlation between Prdx1 and PCNA (Fig. 2A), indicating a possible role of Prdx1 in cell proliferation. To investigate the impact of Prdx1 on OSCC progression, we performed a loss-of-function experiment using siRNA specific to Prdx1. A notable reduction in Prdx1 was observed in siRNA groups, but not in the negative control (NC) group (Fig. 2B). Based on the lowest Prdx1 expression, we selected siRNA1 for SCC15 and CAL27 transfection, with NC-siRNA serving as an NC group.

**Fig. 3:**
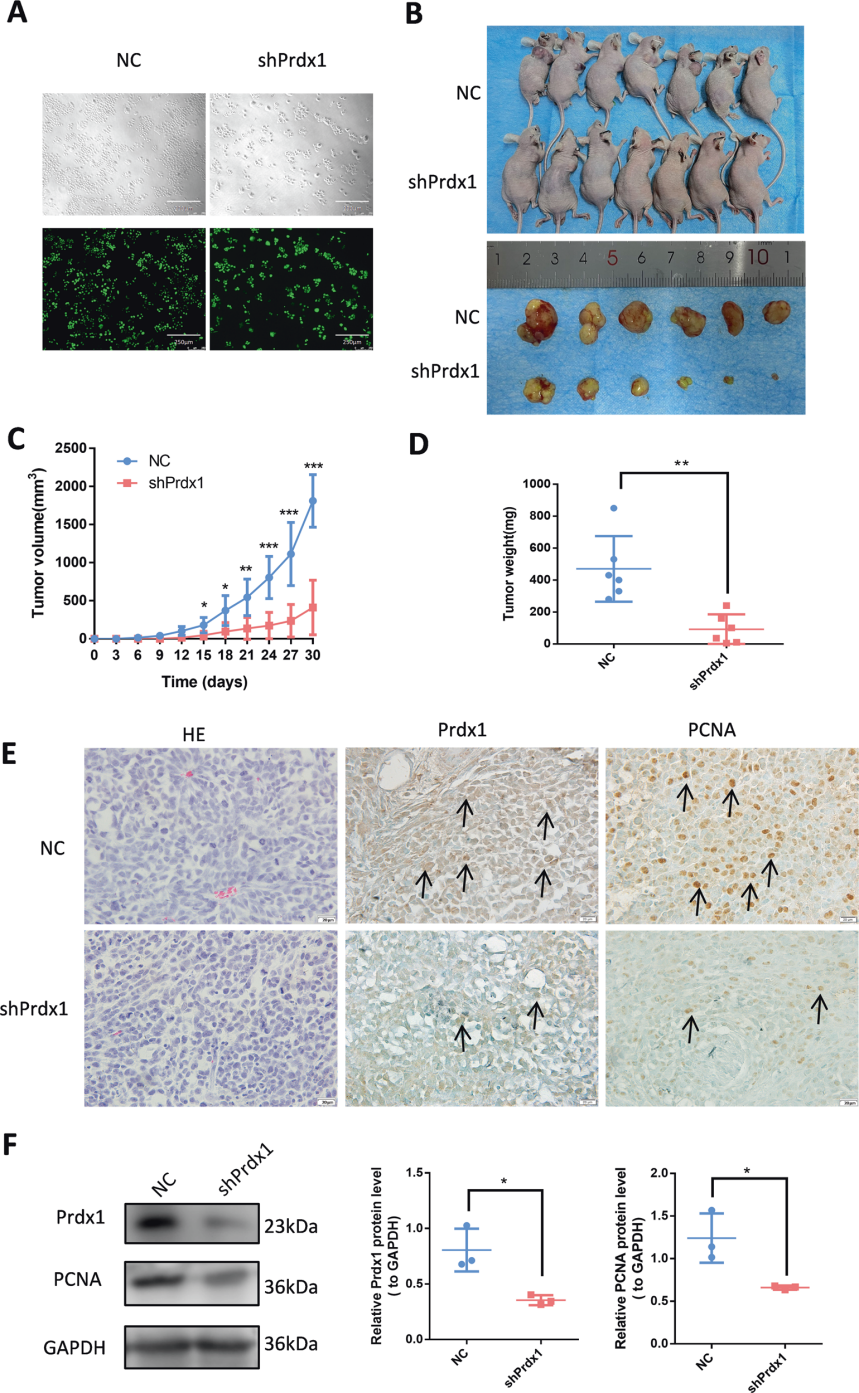
Prdx1 knockdown inhibits the proliferation of CAL27 cells in vivo. **A** Quantification of gene transfer efficiency in CAL27 cells was measured by the proportion of fluorescent cells. **B** The tumorigenicity of endogenous Prdx1 knockdown in CAL27 cells was determined by subcutaneous tumorigenicity test (at day 30) in nude mice (*n* = 7). **C**, **D** The tumor size (from day 0 to day 30) and tumor weight (at day 30) were compared in groups. **E** HE staining and IHC staining of Prdx1 and PCNA (black arrow) in tumor tissue. **F** Western blot analysis of Prdx1, PCNA, and GAPDH in tumor tissues. The data in the bar graph are presented as mean ± SD. *p < 0.05, **p < 0.01, ***p < 0.001.

**Fig. 4:**
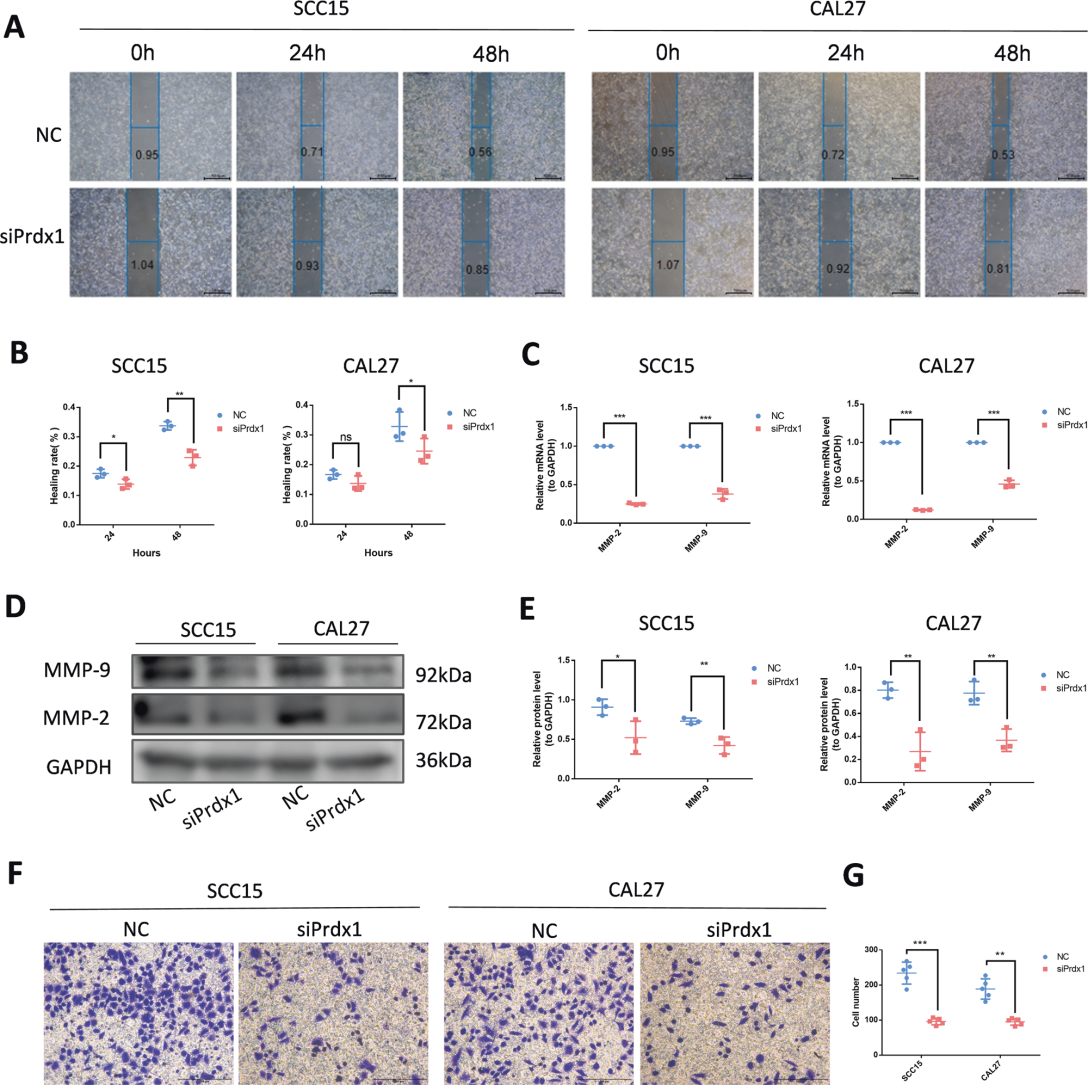
Prdx1 knockdown inhibits the migration of OSCC cells. **A**, **B** Wound healing assay in NC and siPrdx1 groups after 24 and 48 h transfection. **C** The mRNA levels of MMP2 and MMP9 were detected by RT-RCR in NC groups and siPrdx1 groups in SCC15 and CAL27. **D**, **E** The protein levels of MMP2 and MMP9 were detected by western blot in NC groups and siPrdx1 groups in SCC15 and CAL27. **F**, **G** Transwell analysis of siPrdx1 compared with NC. The scale bar was 200 µm. The results are shown as the mean ± SD (*n* ≥ 3), ns, non-significant difference; *p < 0.05; **p < 0.01; ***p < 0.001.

The CCK-8 demonstrated that the proliferation rate of siPrdx1-transfected OSCC cells was significantly reduced compared to the NC group (Fig. 2C, E). A colony formation assay demonstrated that Prdx1 knockdown could decrease the number of foci formed by OSCC cells (Fig. 2D, F). Western blot analysis revealed a significant decrease in PCNA expression in the siPrdx1 group (Fig. 2G). Furthermore, we employed short hairpin RNA (shRNA) to knockdown Prdx1 for subcutaneous tumorigenicity assessment in vivo using a known efficient sequence. Gene transfer efficiency in OSCC cells was quantified by the proportion of fluorescent cells (Figs. 3A, S1A). In the in vivo assay, a significant reduction in tumor volume and weight was observed 15 days post-subcutaneous injection with transfected OSCC cell lines (Figs. 3B–D, S1B–D). IHC and western blot analyses revealed lower Prdx1 and PCNA expression in tumors from the Prdx1-knockdown group compared to the NC group (Figs. 3E, F, S1E, F). Wound healing assay results indicated that a decrease in Prdx1 reduced migration in OSCC cells (Fig. 4A, B). Both mRNA and protein levels of migration-related factors, MMP2 and MMP9, were significantly decreased in the siPrdx1 group compared to the NC group (Fig. 4C–E). Furthermore, transwell assays were performed and the results also showed that knocking down endogenous Prdx1 significantly reduced the number of migrating cells (Fig. 4F, G).

### Prdx1 knockdown inhibits the proliferation and migration of OSCC cells via ROS-mediated pathway

**Fig. 5:**
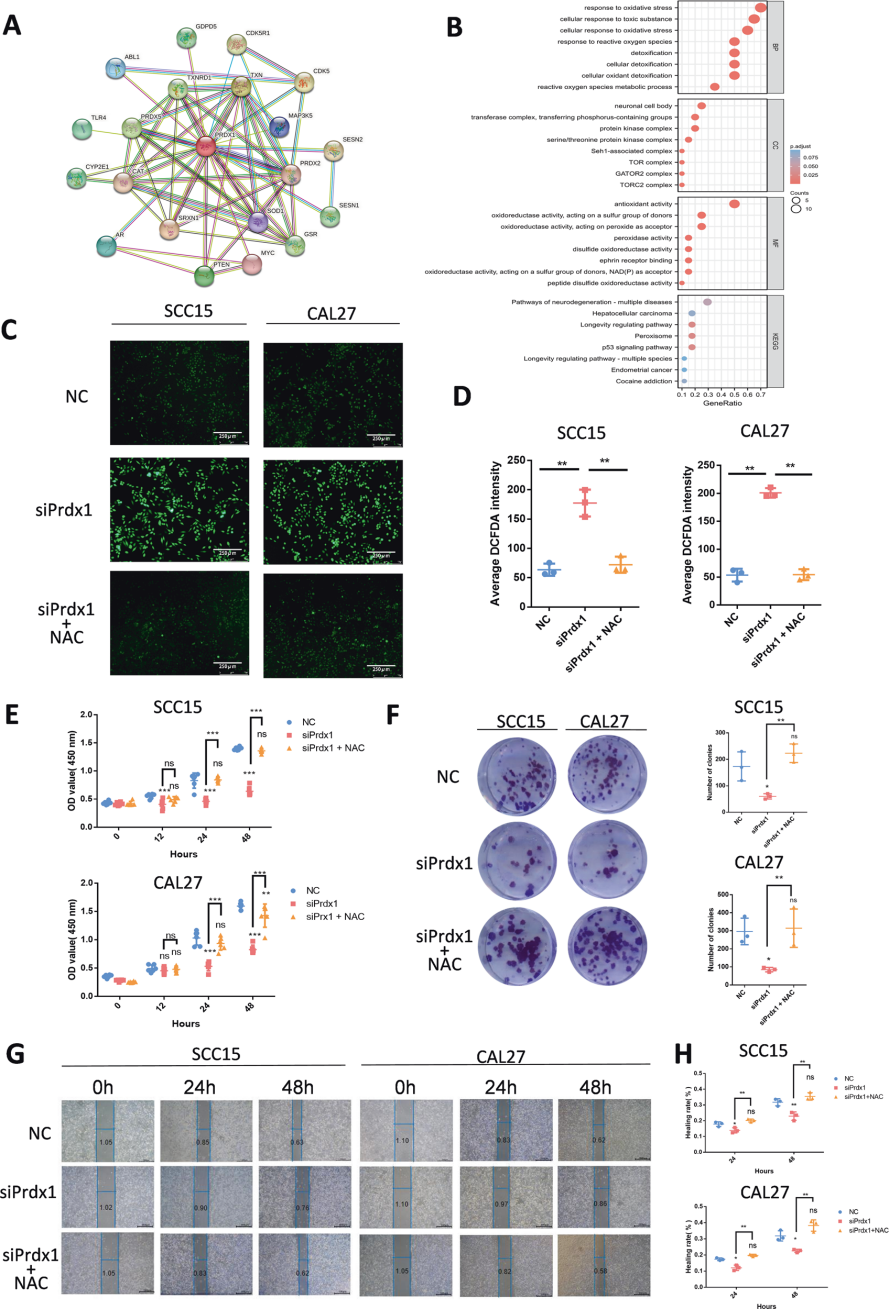
Prdx1 knockdown inhibits the proliferation and migration of OSCC cells via ROS-mediated pathway. **A** STRING protein network interaction analysis. **B** GO term aspects and KEGG pathways enriched by Prdx1-correlated genes. **C**, **D** ROS detection of NC, siPrdx1, and siPrdx1 plus NAC groups after 24 h transfection. **E**, CCK8 assay in NC, siPrdx1, and siPrdx1+ NAC groups after 0, 12, 24, and 48 h transfection. **F**, Colone formation in NC, siPrdx1, and siPrdx1 plus NAC groups for 14 days. **G**, **H** Wound healing assay in NC, siPrdx1, and siPrdx1 plus NAC groups after 24 and 48 h transfection. The results are shown as the mean ± SD (*n* ≥ 3), ns, non-significant difference; *p < 0.05; ** *p* < 0.01; ***p < 0.001.

To elucidate the interconnections among the top 20 PRDX1-related genes, a protein-protein interaction (PPI) network was generated using the STRING online database (Fig. 5A, Table 3). Bubble charts were employed to represent the enrichment results concerning three Gene Ontology (GO) categories and Kyoto Encyclopedia of Genes and Genomes (KEGG) pathways associated with PRDX1-correlated genes (Fig. 5B). These visualizations revealed that PRDX1-correlated genes predominantly participate in oxidative stress and ROS-related biological processes and signaling pathways. Previous research has established that Prdx1 is involved in cellular functions via both ROS-dependent and ROS-independent signaling pathways [8]. Consequently, we investigated whether ROS played a role in the regulatory mechanism of Prdx1. N-acetylcysteine (NAC), a ROS scavenger, was introduced to the siPrdx1 groups. DCFH-DA staining in transfected cells was substantially elevated compared to NC cells but was counteracted by NAC pretreatment (Fig. 5C, D). As shown in the CCK8 and colony formation assay, the siPrdx1+NAC group exhibited a significant recovery in proliferation capacity and colony-forming efficiency compared to the siPrdx1 group (Fig. 5E, F). The wound healing assay revealed that NAC counteracted the anti-migration effect induced by Prdx1 knockdown, with no notable difference observed between the siPrdx1+NAC and NC groups (Fig. 5G, H). Collectively, these findings suggest that ROS plays a role in mediating the impact of endogenous Prdx1 on the biological behavior of OSCC cells.

### Prdx1 knockdown promotes pyroptosis via ROS-dependent pathway and inhibits autophagy via ROS-independent mechanism in OSCC cells

**Fig. 6:**
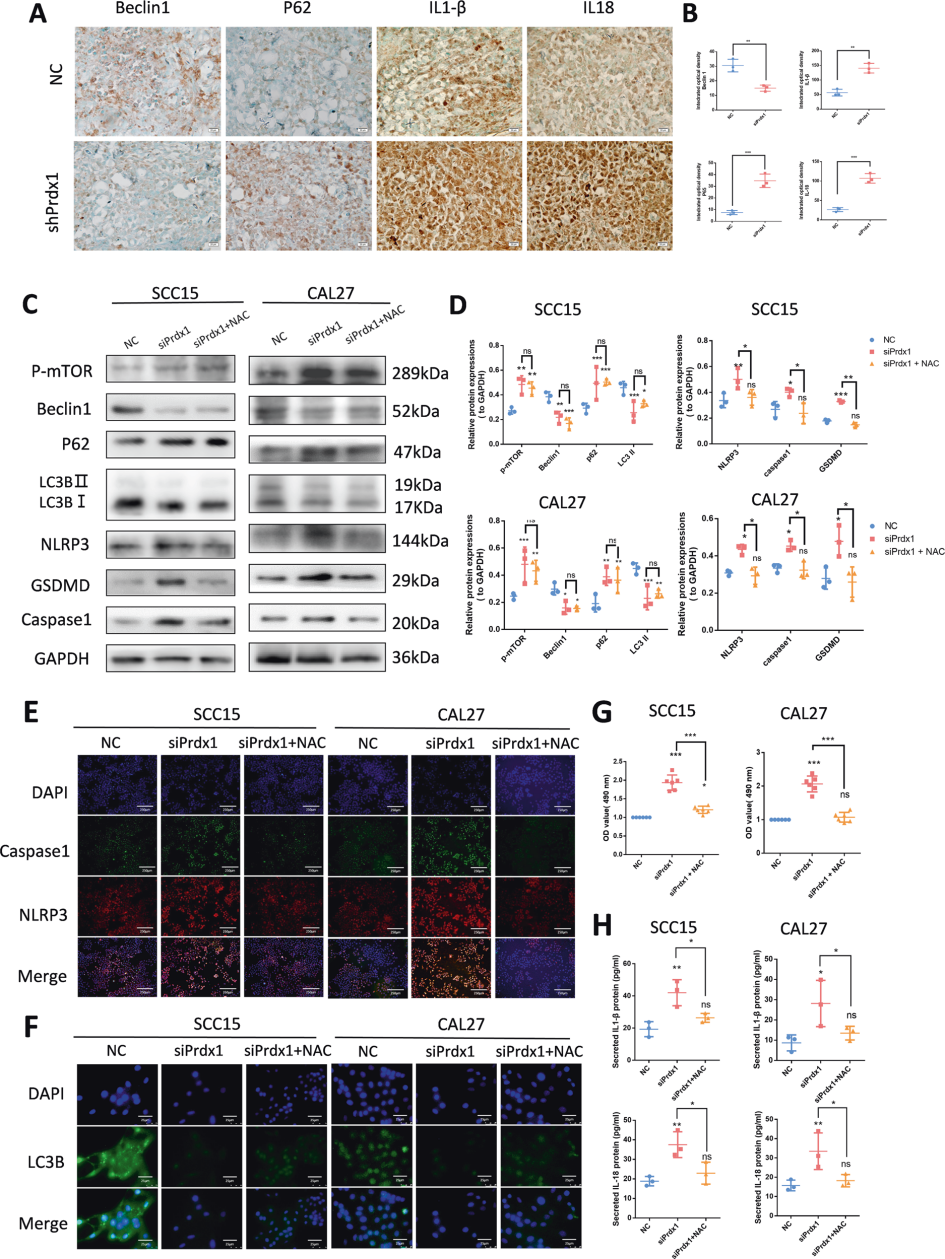
Prdx1 knockdown promotes pyroptosis via a ROS-dependent pathway and inhibits autophagy via a ROS-independent mechanism in SCC15 cells and CAL27 cells. **A**, **B** IHC staining of pyroptosis-related proteins and autophagy-related proteins of tumor tissues in NC and siPrdx1 group. **C**, **D** Western blot showed that the pyroptosis-related and autophagy-related protein levels in NC, siPrdx1 group, and siPrdx1 plus NAC group; Grayscale analysis of each western blotting bands were normalized and quantified with internal controls (GAPDH). **E** Immunofluorescence of pyroptosis-related proteins in NC, siPrdx1 group and siPrdx1 plus NAC group. **F** Immunofluorescence of autophagy-related proteins in NC, siPrdx1 group and siPrdx1 plus NAC group. **G** LDH release assay in NC, siPrdx1 group, and siPrdx1 plus NAC group. **H** ELIZA of IL1-β, IL-18 in NC, siPrdx1 group and siPrdx1+ NAC group. The results are shown as the mean ± SD (*n* ≥ 3), ns, non-significant difference; *p < 0.05; ** *p* < 0.01; ***p < 0.001.

To further elucidate the mechanism of siPrdx1-induced cell death, the level of pyroptosis and autophagy were measured with methods such as WB, IHC, IF, and ELISA. At the tissue level, IHC analysis showed an increase in IL1-β and IL-18 (Figs. 6A, B, S1G, H). At the cellular level, western blotting revealed increased expression of NLRP3 (144kD) and elevated cleavage levels of GSDMD (29kD) and caspase-1 (20kD) in siPrdx1 groups compared to the control groups (Fig. 6C, D). Similar results were obtained with IF (Fig. 6E). We also assessed the release of LDH with an LDH assay kit, and IL1-β, and IL-18 with ELISA to reflect the membrane integrity. siPrdx1-treated cells displayed more than a 1.5-fold increase in LDH release rate (Fig. 6G). Analogous to its upstream factors, secretions of IL1-β and IL-18 were elevated (Fig. 6H). A reduction in pyroptosis-related proteins and LDH release was observed when Prdx1-silenced cells were pretreated with NAC (Fig. 6C, E, and G). These results suggest that Prdx1 knockout induces pyroptosis via the NLRP3/GSDMD/caspase-1 axis in OSCC and that ROS scavenging reverses siPrdx1-induced pyroptosis. Similar alterations of autophagy, such as Beclin1 and P62, were observed in the IHC and WB experiments (Figs. 6A–D, S1G, H). In comparison to the control group, the siPrdx1 group exhibited decreased Beclin1 and LC3B-II levels and increased p-mTOR and p62 levels (Fig. 6C, D, and E). Visualization of LC3B puncta is considered the gold standard for assessing autophagy induction [20]. Immunofluorescence results showed enhanced intensity of LC3B and mature puncta, indicative of normal autophagic vacuoles, in control cells, whereas diffuse green fluorescence was observed following siRNA treatment (Fig. 6F). No significant changes in autophagic protein levels nor discernible alterations in LC3B puncta size and number were detected in OSCC cells transfected with siPrdx1, regardless of NAC treatment (Fig. 6C, F). These findings indicate that Prdx1 knockdown inhibits autophagy in OSCC cells through a ROS-independent mechanism.

### Prdx1 regulates crosstalk between pyroptosis and autophagy in OSCC cells

**Fig. 7:**
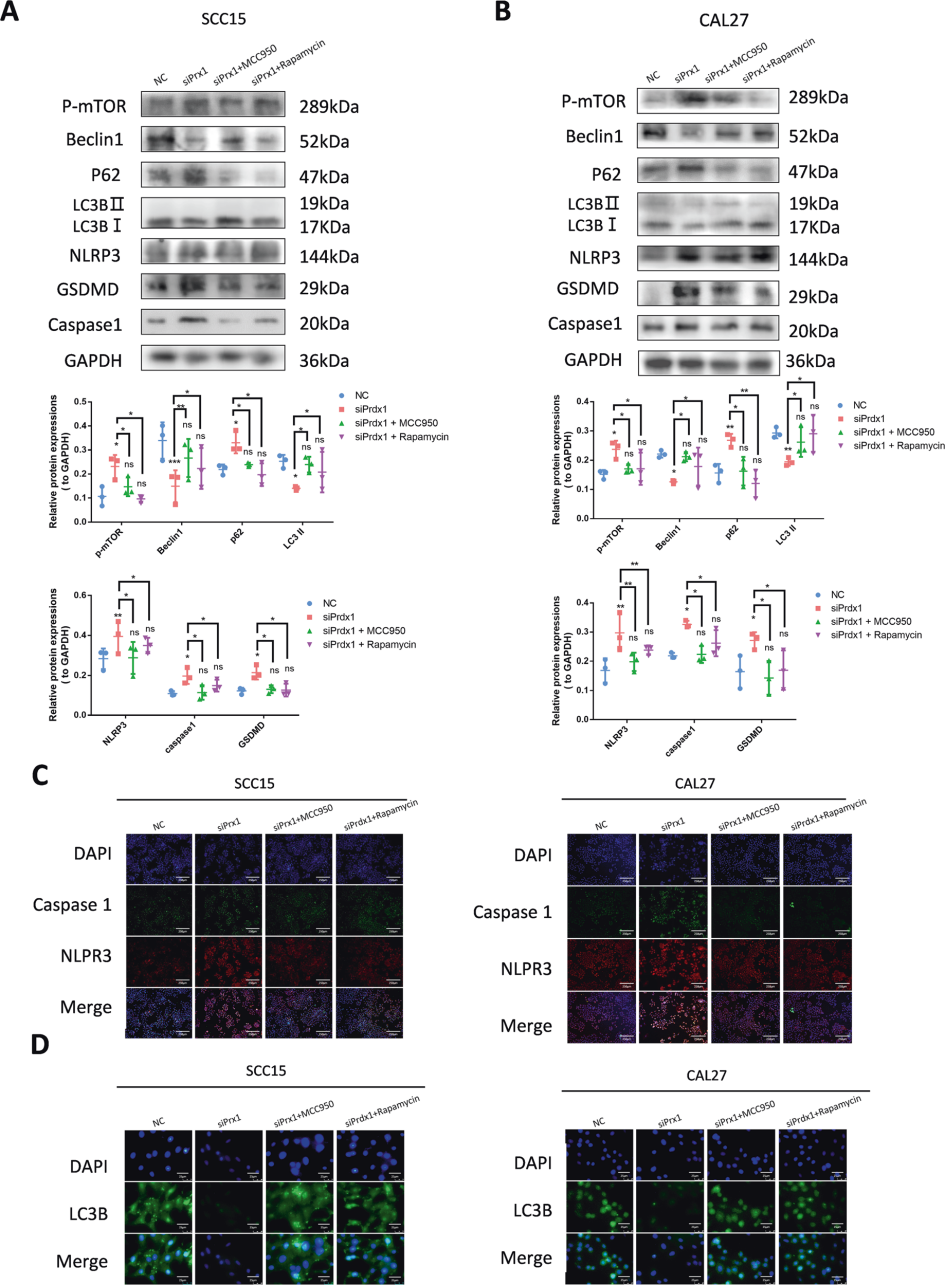
Prdx1 regulates crosstalk between pyroptosis and autophagy in OSCC cells. **A**, **B** Western blot and grayscale analysis showed that the pyroptosis-related and autophagy-related protein levels in NC group, siPrdx1 group, siPrdx1 plus MCC950 group, and siPrdx1 plus Rapamycin group. **C** Immunofluorescence of pyroptosis-related proteins in NC group, siPrdx1 group, siPrdx1 plus MCC950 group, and siPrdx1 plus Rapamycin group. **D** Immunofluorescence of autophagy-related proteins in NC group, siPrdx1 group, siPrdx1 plus MCC950 group, and siPrdx1 plus Rapamycin group. The results are shown as the mean ± SD (*n* ≥ 3), ns, non-significant difference; *p < 0.05; **p < 0.01; ***p < 0.001.

An increasing number of studies have highlighted the intricate relationship between pyroptosis and autophagy [21–26]. We further investigated the involvement of endogenous Prdx1 in this interaction within OSCC cells. We observed that pretreatment with MCC950, a specific NLRP3 inflammasome inhibitor, significantly reduced the levels of NLRP3 induced by siPrdx1, as well as its downstream effectors caspase-1 and GSDMD (Fig. 7A, B). Interestingly, this restored the autophagic factors that were suppressed by siPrdx1 transfection. There were notable differences in the protein levels of mTOR and p62 between the siPrdx1 and siPrdx1 + MCC950 groups, further evidenced by the reestablishment of Beclin1 and LC3 conversion (Fig. 7A, B), and the reappearance of distinct LC3B puncta in immunofluorescence (Fig. 7D). Conversely, when employing rapamycin as a specific inhibitor of mTOR phosphorylation, we observed a considerable decrease in NLRP3, caspase-1, and GSDMD protein levels and fluorescent intensity between the siPrdx1 and siPrdx1+rapamycin groups, accompanied by the resurgence of pro-autophagic markers (Fig. 7A–C). We inferred that rapamycin treatment restored the autophagy suppressed by the knockdown of endogenous Prdx1 and substantially eliminated NLRP3 expression, caspase-1 maturation, and GSDMD cleavage induced by endogenous Prdx1 knockdown. In summary, the inhibition of pyroptosis enhanced the autophagy suppressed by siPrdx1, while the activation of autophagy counteracted the pyroptosis induced by siPrdx1 in OSCC cells. Endogenous Prdx1 plays a pivotal role in modulating the interplay between autophagy and pyroptosis in OSCC cells.

## Discussion

In this study, we investigated the expression and biological functions of Prdx1 in OSCC. Our results indicated that Prdx1 is overexpressed in tumor tissues and cell lines of OSCC and plays a crucial role in supporting cell proliferation and mobility by reducing excessive ROS. Prdx1 may serve as a link between pyroptosis and autophagy in OSCC.

Prdx1 is an antioxidant enzyme that possesses a robust capacity for maintaining intracellular redox equilibrium. Although its role in diseases such as inflammation and cancer has been gradually elucidated as research has deepened, there have been a few studies exploring the expression and mechanism of Prdx1 in OSCC. In OSCC, increased levels of Prdx1 have been observed in previous studies [7, 12], while contrasting findings have also been reported [27]. Prdx1 is elevated in oral leukoplakia and dysplastic oral keratinocytes and is crucial for the transformation of precancerous lesions into malignant cancer [28, 29]. By analyzing Prdx1 mRNA data from TCGA-HNSC, we discovered that Prdx1 expression was significantly upregulated in HNSC samples compared to normal samples, and negatively correlated to survival. This upregulation was further confirmed in OSCC tumor tissues and cell lines through IHC, western blot, and RT-PCR analyses.

Active cell proliferation and mobility are fundamental characteristics of malignant cells that contribute to tumor development and progression. In esophageal squamous cell carcinoma (ESCC), Prdx1 silencing has been shown to inhibit cell proliferation and induce apoptosis, whereas Prdx1 overexpression produces growth-promoting effects [11, 30]. Niu et al. demonstrated that Prx1 is essential for nicotine-induced cell invasion and migration via the epithelial-to-mesenchymal transition (EMT) process in OSCC cells [12]. Aberrant Prdx1 expression and its impact on the malignant phenotypes have not been well studied in OSCC. In this study, IHC results demonstrated a concurrent upregulation of Prdx1 and PCNA proteins in OSCC tissues compared to adjacent non-cancerous tissues. Consistently, bioinformatic analysis revealed a significant positive correlation between Prdx1 and PCNA expression. Following Prdx1 knockdown, we observed a substantial decrease in cell viability and colony numbers in vitro, as well as a reduction in the size and weight of xenografts. Our findings indicate that Prdx1 deficiency results in diminished PCNA expression both in vitro and in vivo. MMP2 and MMP9 were crucial in EMT and tumor invasion. In our study, we obtained direct evidence that knockdown of Prdx1 impeded cell migration and invasion and reduced the expression of MMP2 and MMP9.

Pyroptosis and autophagy are two different forms of PCD and are implicated in various diseases, including inflammations and cancers. There is accumulating evidence that pyroptosis is associated with proliferation, metastasis, and invasion in various cancers [13]. In recent years, drugs targeting pyroptosis have shown promise in cancer treatment [31]. Alpinumisoflavone (AIF) has been found to induce NLRP3 inflammasome assembly and pyroptosis activation, exerting anti-proliferative and anti-metastatic effects; these effects were reversed by NLRP3 shRNA and MCC950, an NLRP3 inhibitor [32]. Taxol and cisplatin were shown to induce pyroptosis through the GSDMD/caspase-1 pathway in nasopharyngeal carcinoma and breast cancer, respectively [33, 34]. Current research on the relationship between the peroxiredoxin family and pyroptosis is limited and inconclusive. Prdx3 has been reported to promote pyroptosis in benign prostatic hyperplasia (BPH) [35] and also act as a suppressor of pyroptosis by inhibiting NLRP3 inflammasome activation [36]. The role of pyroptosis in OSCC remains underexplored. Our results demonstrated that Prdx1 deficiency induced pyroptosis, as evidenced by increased NLRP3 expression, caspase-1 and GSDMD cleavage, and LDH release. Prior research has demonstrated autophagy’s involvement in OSCC proliferation, angiogenesis, and resistance [16, 37–39], suggesting that modulating autophagy may offer a novel direction for OSCC therapeutic strategies. The mechanism by which Prdx1 modulates autophagy in OSCC remains to be determined. In our study, siPrdx1 treatment resulted in increased mTOR phosphorylation and p62 expression, while reducing Beclin1 expression and LC3B conversion. Immunofluorescence images revealed diffuse LC3B fluorescence, indicating impaired autophagosome formation.

ROS is suggested as an important participator in the occurrence and development of tumors. Emerging evidence indicates that Prdx1 participates in cellular activities through both ROS-dependent and ROS-independent signaling pathways [8]. Excessive ROS accumulation is fetal for cancer cells and considered as an approach for treating cancers, such as the mediator of radiotherapy and chemotherapy for damaging DNA of cancer cells. Prdx1 was reported to enable cancer cells to withstand high ROS stress and acquire resistance to radio- and chemotherapy [40]. In our study, intracellular ROS levels increased upon Prdx1 knockdown. We observed a recovery of proliferation and migration when we introduced NAC as a ROS scavenger in the siPrdx1 group, suggesting that endogenous Prdx1 enhances the proliferation and migration of OSCC cells through a ROS-suppressed mechanism. It is important to highlight the complex interplay between ROS, pyroptosis, and autophagy at multiple stages. In lung cancer, Liu J et al. demonstrated that ROS generation contributes to NLRP3 activation [41], and Zhu M et al. reported that GSDMD initiation was driven by ROS acceleration, both of which ultimately resulted in pyroptotic cell death [42]. Autophagy serves as a protective mechanism against cell death from ROS injury. Baicalein was shown to induce both apoptosis and autophagy through ROS generation, and the pharmacological or genetic inhibition of autophagy enhanced the anticarcinogenic activity of baicalein in OSCC by promoting apoptosis [43]. In our study, ROS scavenging reversed siPrdx1-induced pyroptosis but had no impact on autophagy. These observations suggest that ROS is involved in Prdx1-triggered pyroptosis but has limited influence on Prdx1-regulated autophagy.

Elucidating the interplay between pyroptosis and autophagy may shed light on novel biological mechanisms and therapeutic approaches. Autophagic clearance of pathogens and the NLRP3 inflammasome may constrain the pyroptotic response [21, 22]. However, exceptions exist, as autophagic inhibition in ovarian cancer cells hindered pyroptosis by downregulating active GSDMD and GSDME [23]. Regarding the influence of pyroptosis on autophagy, necrosulfonamide (NSA) treatment, a specific GSDMD inhibitor, resulted in diminished LC3II expression in ovarian cancer cells [23]. Another study demonstrated that pyroptosis negatively regulated autophagy, while NLRP3 inhibition enhanced autophagy through the suppression of the mTOR signaling pathway [24]. Researchers have investigated potential mediators in this interaction. In a prion disease model, both pyroptosis and autophagy occurred in PrP106–126-treated microglia via the TLR4-TRIF pathway. Caspase-1-mediated cleavage of the TRIF segment partially explained the inhibitory effect of the inflammasome on autophagy [25]. Furthermore, caspase-1 hindered mitophagy by cleaving Parkin, thereby exacerbating mitochondrial damage [26]. In our study, we observed a crosstalk between pyroptosis and autophagy in OSCC cells, where pyroptosis inhibition partially restored siPrdx1-suppressed autophagy, while autophagy activation attenuated siPrdx1-induced pyroptosis. We used MCC950 as a highly selective NLRP3 inhibitor to downregulate NLRP3 and downstream factors and subsequently observed autophagy recovery and autophagosome repossession. By inhibiting mTOR with rapamycin, we induced autophagy in Prdx1-silencing cells. Rapamycin apparently attenuated pyroptosis-related protein activation.

To the best of our knowledge, our study represents the first exploration of the impact of Prdx1 on pyroptosis and the innovative examination of the reciprocal relationship between pyroptosis and autophagy in OSCC. Further comprehensive investigations are necessary to unveil the intricate molecular mechanisms underlying the involvement of Prdx1 in cellular processes and the crosstalk between pyroptosis and autophagy. Consequently, substantial progress must be made before Prdx1 can be considered a viable therapeutic target in a clinical setting.

## Conclusion

**Fig. 8:**
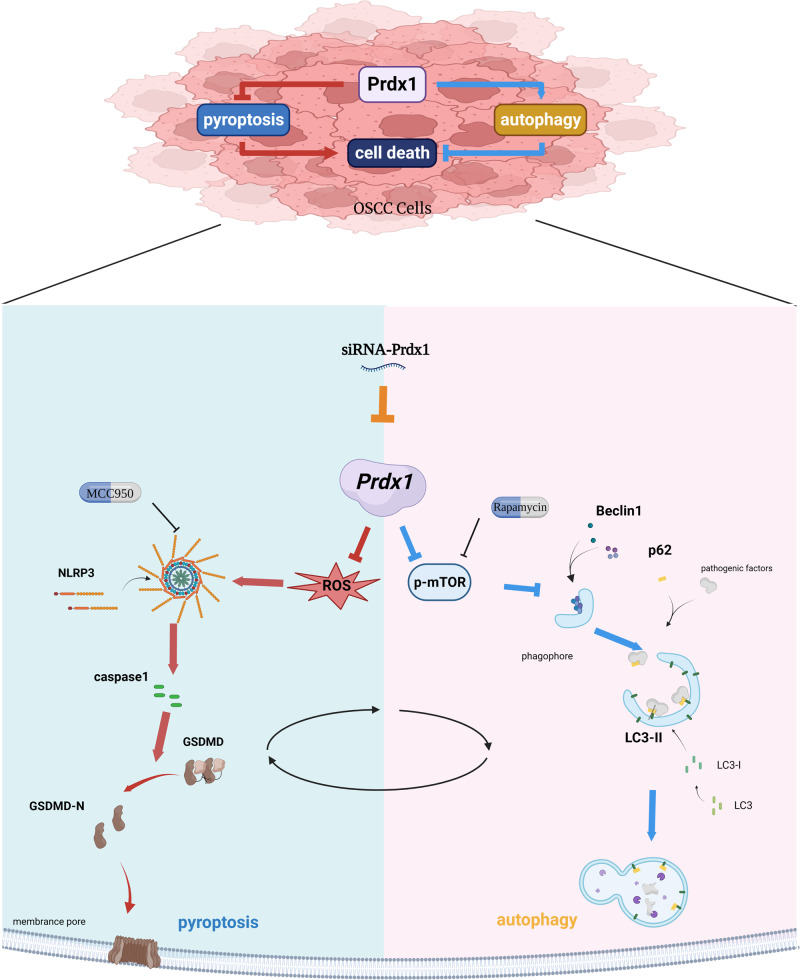
Model diagram of Prdx1 regulating OSCC cells. Endogenous Prdx1 in OSCC cells modulates the disease progression through the inhibition of autophagy via a ROS-independent mechanism, promotion of pyroptosis via a ROS-dependent mechanism, and involvement in the intricate interplay between autophagy and pyroptosis.

In summary, our study has elucidated the crucial role of Prdx1 overexpression in the progression of OSCC. We demonstrated that the silencing of endogenous Prdx1 in OSCC cells modulates the disease progression through the inhibition of autophagy via a ROS-independent mechanism, promotion of pyroptosis via a ROS-dependent mechanism, and involvement in the intricate interplay between autophagy and pyroptosis (Fig. 8). Collectively, these insights highlight the potential of Prdx1 as a promising therapeutic target for the treatment of OSCC patients.

## Methods and materials

### Human tissue samples

Paraffin tissue sections of 20 OSCC patients were obtained from the Department of Pathology, Shandong Provincial Stomatological Hospital. In our study, all patients were diagnosed by pathology, and no other treatments such as radiotherapy or chemotherapy before surgery. The study has been approved by the Bioethics Committee of Shandong Stomatological Hospital (Approval No. 20221002) and all patients provided informed consent.

### Bioinformatics analysis

RNA-sequencing data of head and neck carcinoma (HNSC) patients were downloaded from the TCGA database (https://portal.gdc.cancer.gov/). The TCGA-HNSC genotype data of normal samples (*n* = 44) and tumor samples (*n* = 502), among which were 43T/N pairs, was analyzed in the R program (version 3.6.3). Survival analysis was generated using Kaplan–Meier plotter (http://kmplot.com). The hazard ratio with 95% confidence intervals and log-rank *p* value were calculated. We used the tumor immune estimation resource (TIMER) database (http://cistrome.dfci.harvard.edu/TIMER/) to analyze the difference in PRDX1 expression between tumor and normal tissues in pan-cancer. The STRING (https://string-db.org) database (version 11.5) provides access to experimental and predicted information concerning the interactions of lists of PRDX1. The gene names of the top 20 PRDX1-related genes (Table 1) were converted to the Entrez ID and analyzed by using the clusterProfiler package (version 3.14.3) in the R program (version 3.6.3). The Benjamini and Hochberg (BH) method was used for calculating the adjusted *p* values. The GO terms including biological process (BP), cellular component (CC), molecular function (MF), and KEGG pathways that were significantly enriched by the correlated genes were identified by setting the threshold of *p*.adj < 0.05 and *q* value < 0.2. All data were visualized by the ggplot2 package (version 3.3.3) in the R program (version 3.6.3).

**Table 1:** Top 20 PRDX1-related genes.

Name	Gene ID	Description	Score
SRXN1	ENSP00000371388	Contributes to oxidative stress resistance	0.998
TXN	ENSP00000363641	Participates in various redox reactions	0.997
PRDX5	ENSP00000265462	Catalyzes the reduction of hydrogen peroxide and organic hydroperoxides to water and alcohols, respectively	0.983
PTEN	ENSP00000361021	Tumor suppressor	0.982
TLR4	ENSP00000363089	Mediates the innate immune response to bacterial lipopolysaccharide (LPS)	0.976
AR	ENSP00000363822	Regulates eukaryotic gene expression and affects cellular proliferation and differentiation in target tissues	0.971
ABL1	ENSP00000361423	Regulates cell growth and survival	0.968
MAP3K5	ENSP00000351908	An essential component of the MAP kinase signal transduction pathway	0.966
MYC	ENSP00000479618	Activates the transcription of growth-related genes	0.962
SOD1	ENSP00000270142	Destroys radicals	0.962
PRDX2	ENSP00000301522	catalyzes the reduction of hydrogen peroxide and organic hydroperoxides to water and alcohol, respectively.	0.945
CAT	ENSP00000241052	protects cells from the toxic effects of hydrogen peroxide	0.937
SESN2	ENSP00000253063	an intracellular leucine sensor that negatively regulates the TORC1 signaling pathway through the GATOR complex	0.926
CDK5	ENSP00000419782	Interacts with D1 and D3-type G1 cyclins.	0.91
CDK5R1	ENSP00000318486	Cyclin-dependent kinase 5 regulatory subunit 1	0.9
TXNRD1	ENSP00000434516	Thioredoxin reductase 1	0.874
GSR	ENSP00000221130	Maintains high levels of reduced glutathione in the cytosol	0.858
SESN1	ENSP00000393762	an intracellular leucine sensor that negatively regulates the TORC1 signaling pathway through the GATOR complex	0.844
GDPD5	ENSP00000337972	Cooperates with PRDX1 to drive postmitotic motor neuron differentiation.	0.842
CYP2E1	ENSP00000440689	Metabolizes several precarcinogens, drugs, and solvents to reactive metabolites.	0.83

### Hematoxylin and eosin (HE) staining and immunohistochemical (IHC) staining

After dewaxing and hydration, the sections were soaked in hematoxylin dye for 15 min and then stained with eosin dye for 7 min. Next, the sections were dehydrated with an ascending alcohol concentration gradient, soaked twice in xylene solution, and last mounted with neutral gum. In order to perform IHC staining, the sections were incubated with 0.3% hydrogen peroxide for 30 min at room temperature and then treated with 1% bovine serum albumin (BSA) in PBS for 20 min at room temperature to prevent non-specific staining. Next, the sections were treated with primary antibodies (anti-Prdx1, 1:100, abcam, ab15571; anti-PCNA, 1:100, calbiochem, NA03; anti-Beclin1, 1:100, Proteintech, 11306-1-AP; anti-P62, 1:100, Proteintech, 18420-1-AP; anti-IL18, 1:100, Proteintech,10663-1-AP; anti-IL1-β, 1:100, Proteintech, 260481-AP) at room temperature. The next day, after rinsing in PBS, sections were subjected to secondary antibody (anti-rabbit, 1:200; anti-mice, 1:200) staining for 1 h at room temperature. Diaminobenzidine (Sigma-Aldrich, Merck KGaA, Germany) was used to examine immune responses. Finally, all the sections were counterstained with methyl green stain before being viewed under an optical microscope (Olympus BX-53, Tokyo, Japan).

### Cell culture

Cells lines SCC15, SCC25, and CAL27, were purchased from the cell banks of the Chinese Academy of Sciences (Shanghai, China). The cells were cultured in DMEM/Ham’s-F12 medium (Gibco, Grand Island, NY, USA) supplemented with 10% fetal bovine serum (FBS; Gibco, Grand Island, NY, USA) and 1% penicillin–streptomycin at 37 °C in a 5% CO_2_ humidified atmosphere.

### Cell Transfection

The siRNAs targeting Prdx1 were designed and synthesized by RiboBio (Guangzhou, China). According to the manufacturer’s protocol, when cells grew up to ~50%, the siRNAs were transfected into cells with the transfection reagent (RiboBio, Guangzhou, China). After 24 h transfection, cells were used to experiment further. In knockdown analysis, at least a 50% reduction in Prdx1 expression is considered an effective transfection. The siRNA sequences are listed in Table 2.

**Table 2:** The siRNA sequences used in the experiment.

Name	Target sequence
genOFFTM st-h-PRDX1_001	CCATGAACATTCCTTTGGT
genOFFTM st-h-PRDX1_002	GGAGATCATTGCTTTCAGT
genOFFTM st-h-PRDX1_003	CAGCCTGTCTGACTACAAA

### CCK-8 assay

OSCC cells were harvested and planted in 96-well plates at the density of 5×10³ cells/well, and grown adherently for 24 h. After being exposed to siPrdx1 for a certain period of treatment (0, 12, 24, 48 h), each well was treated with a 10 μl cell counting kit −8 (CCK-8) reagent (HYK0301; MedChemExpress) and incubated at 37 °C for 1.5 h. An enzyme-labeling instrument detected the absorbance at 450 nm by an enzyme-labeling tool (iMark, Bio-Rad Laboratories, Inc.). Data was exported as Excel files and was analyzed with GraphPad Prism 6.00 (San Diego, CA, USA). With six independent repeats in each group, growth curves were drawn in the form of mean ± standard error of the mean.

### Colony formation assay

We planted cells of three cell lines (500 cells/well) into 12-well plates. After 24 h, cells were exposed to different treatments and incubated for 14 days until visible clones could be seen. They were fixed with 4% paraformaldehyde (PFA), dyed with 0.1% crystal violet, and photographed under an optical microscope (Olympus BX53, Tokyo, Japan). The number of colonies was counted by ImageJ, and analyzed by GraphPad Prism 6.00. Statistics of three repetitions were presented as bar graphs in the form of mean ± standard error of the mean.

### In vivo tumorigenicity assay

Lentiviral vector delivery of short hairpin RNA (shRNA), constructed by Keyybio (Shandong, China), was used to knockdown Prdx1 (shPrdx1) for tumorigenicity assay in vivo. SCC15 and CAL27 cells (8 × 10^4^ cells) were prepared and then infected at a multiplicity of infection (MOI) of 20 or empty lentiviral vectors with a green fluorescent protein. After 24 h incubation, the cells were supplemented with fresh serum medium. Then OSCC cells were selected with puromycin (SCC15 5 μg/ml, CAL27 2 μg/ml; 5 days; Solarbio, Beijing, China) for stable transfection. In order to identify the efficiency of lentivirus infection, OSCC cells were observed under a fluorescent microscope. 28 SPF-grade BALB/c nu/nu male nude mice (6 weeks old) without a specific pathogen were used. This study was approved by the Bioethics Committee of Shandong Stomatological Hospital (Approval No. 20221003). The nude mice were randomly divided into the negative control group and shPrdx1 group with 7 mice in each group after one week of adaptive feeding. Then, 0.1 ml of NC and shPrdx1 OSCC cell suspensions at a concentration of 1 × 10^7^ cells/ml were subcutaneously injected into the right back of each mouse. Tumor volumes (volume = length × width^2^/2) were measured every 3 days from 7 days after injection until 30 days. The tumor tissues were divided into two parts, one was analyzed by western blot and the other was cut into 5 µm sections. Sections were analyzed by HE staining or IHC staining.

### Wound healing assay

Cells were plated into six-well plates and cultured 24 h adherently before transfection. After transfection for about 36 h, when cell density came up to 80–90%, we scratched a linear wound on the cell layer with a 200 μl-pipet tip. The cells were washed with PBS and were incubated in a serum-free medium. Selected sites on plates were photographed under the optical microscope at 0, 24, and 48 h. Scratched distances were measured by ImageJ. Utilizing GraphPad Prism 6.00, the ability of migration was estimated by healing rate (original distance−real-time)/original distance*100%, and statistical charts were drawn in the form of mean ± standard error of the mean.

### Transwell assay

After 24 h of transfection, 3 × 10^4^ cells in 100 µl serum-free medium were added to the upper chamber of the 24-well plate, and the lower chamber contained 600 µl of culture medium with 10% FBS. The pore size of transwells was 0.22 µm. After a period of 48 h, we wiped out the cells that had not migrated through the membrane and fixed the migrated cells in 4% paraformaldehyde for 30 min and then stained them with 0.1% crystal violet for another 20 min. The cells were photographed with a light microscope (Olympus BX53, Tokyo, Japan) and counted for analysis.

### Detection of reactive oxygen species

Cells were seeded in 24-well plates with 2 × 10^4^ cells per well. After treatment, a 10 μm dichloro-dihydro-fluorescein diacetate (DCFH-DA) fluorescent probe in the fresh medium was added to the wells at 37 °C for 30 min. Then the plates were submitted to a fluorescence microscope (Leica, Germany). Fluorescence intensity was measured by Image J Launcher.

### Quantitative reverse transcription PCR

After treatments, total cellular RNA was extracted by Trizol reagent (AG21102; Accurate Biotechnology (Human) Co., Ltd.). According to the manufacturer’s protocol, the Evo M-MLV RT Reverse Transcription Kit II (AG11711; Accurate Biotechnology. (Human) Co., Ltd.) was used to reverse transcript RNA into cDNA in the condition of 37 °C 15 min and 85 °C 5 s. We sent cDNA to a real-time polymerase chain reaction (RT-PCR) detection system (Heal Force, Shanghai, China) with the help of the SYBR green I Mix (Accurate Biology, Hunan, China). One thermal cycle of reaction composed of 95 °C 5 s, 55 °C 10 s, and 72 °C 15 s, 40 cycles were performed. Data was analyzed with GraphPad Prism 6.00. GAPDH mRNA acted as an internal reference to normalize the relative expression of the targeted gene. Fold changes of expression were calculated by the 2^−∆∆CT^ method. The primer sequences used in the experiment are listed in Table 3.

**Table 3:** The primer sequences used in the experiment.

Name	Forward primer	Reverse primer
GAPDH	CCTGCACCACCAACTGCTTA	GGCCATCCACAGTCTTCTGAG
Prdx1	AGCTGTTATGCCAGATGGTCAG	CACCAATCACTTGGCAGTTGAG
MMP2	CTCATCGCAGATGCCTGGAA	TTCAGGTAATAGGCACCCTTGAAGA
MMP9	ACGCACGACGTCTTCCAGTA	CCACCTGGTTCAACTCACTCC

### Western blot analysis

After different treatments, total cellular proteins were extracted with a mixture composing RIPA lysis buffer (Beyotime, Shanghai, China), 1% protease, and 1% phosphatase inhibitors. The protein concentrations were measured by BCA assay (Beyotime, Shanghai, China). Equal protein amounts (30 μg) were loaded on sodium dodecyl sulfate–polyacrylamide gel electrophoresis (SDS–PAGE) and then transferred to polyvinylidene fluoride (PVDF) membranes. The membranes were incubated overnight with primary antibodies including rabbit anti-GAPDH (1:2500, Proteintech,10494-1-AP), anti-Prdx1 (1:1000, Abcam, ab15571), anti-MMP2 (1:1000, Merck, MAB3308), anti-MMP9 (1:1000, Abcam, ab283575), anti-caspase-1 (1:1000, Abcam, ab1872), anti-GSDMD (1:1000, Abcam, ab210070), anti-NLRP3 (1:500, Abcam, ab214185), anti-p62 (1:1000, Proteintech, 11306-1-AP), anti-beclin1 (1:1000, Abcam, ab302669), anti-p-mTOR (1:1000, Abcam, ab109268), anti-LC3B (1:1000, Abcam, ab48394) and then followed by the incubation with the secondary antibodies (anti-rabbit 1:2000, Abcam, ab6721; anti-mice 1:2000, CellSignaling, #7076) at room temperature for 1 h. Images were captured by the gel imaging system (Amersham Imager 600; General Electric Company) with the enhanced chemiluminescence (ECL) kit. Values of grayscale were measured by ImageJ.

### Immunofluorescence

Cells were grown on 12-well plates. After the indicated treatments, cells were fixed with 4% PFA for 20 min. Permeabilized by 0.5% Trixon-100, the cells were blocked in 5% BSA–PBS for 1 h at room temperature. Then the plates were placed in 4 °C 12 h with the incubation of specific antibodies including anti-NLRP3 (1:200, Abcam), anti-caspase-1 (1:200, Abcam), anti-LC3B (1:200, Abcam), following by incubation of fluorescent secondary antibodies at 37 °C for 1 h, including fluorescein (FITC)-conjugated Affinipure Goat Anti-Rabbit (1:100, proteintech), rhodamine (TRITC)-conjugated Goat Anti-Mouse (1:100, proteintech). Nuclei were stained in 4’,6’-diamidino-2-phenylindole (DAPI) for 5 min at 25 °C. The cells were observed and shot under the fluorescence microscope.

### LDH release assay

OSCC cells were harvested and planted in 96-well plates at the density of 5 × 10^3^ cells/well, and grown adherently for 24 h. Cells were exposed to siPrdx1 for 24 h, and then 120 μl of supernatant from each hole was added to the corresponding hole of a new 96-well plate. 60 μl LDH detection working liquid was added into each hole and mixed well. Plates were incubated at room temperature for 30 min without light. Absorbance was monitored at 490 nm to quantify LDH concentrations.

### Enzyme-linked immunosorbent assay (ELISA)

The levels of IL1-β and IL18 in the culture medium of each group were detected by ELISA. The tumor cells were incubated in a culture medium containing siRNA for 24 h and then changed to a serum-free culture medium for 24 h before the culture medium was collected. The levels of IL1-β and IL-18 were measured using a cytokine-specific ELISA kit (IL1-β, Proteintech; IL18, Arigo), according to the manufacturer’s instructions.

### Statistical analyses

All experimental data were derived from at least three independent repeats. Data were processed by either an unpaired Student’s *t*-test or one-way ANOVA on GraphPad Prism 6.00 and described as mean ± standard error of the mean. *p < 0.05, **p < 0.01, ***p < 0.001.

### Supplementary information

Supplemental Fig. 1

Supplemental Fig. 1 legend

Original Images for Blots and Gels Requirements

## Data Availability

All data used to support the findings of this study are included within the article. The analyzed data during the current study are available from the corresponding author upon reasonable request.
