# Supplementary figures and images for: Peroxiredoxin 1 regulates crosstalk between pyroptosis and autophagy in oral squamous cell carcinoma leading to a potential pro-survival
Source: Cell Death Discov. 2023 Nov 25;9:425. doi: 10.1038/s41420-023-01720-7 (PMC10676359; doi:10.1038/s41420-023-01720-7)

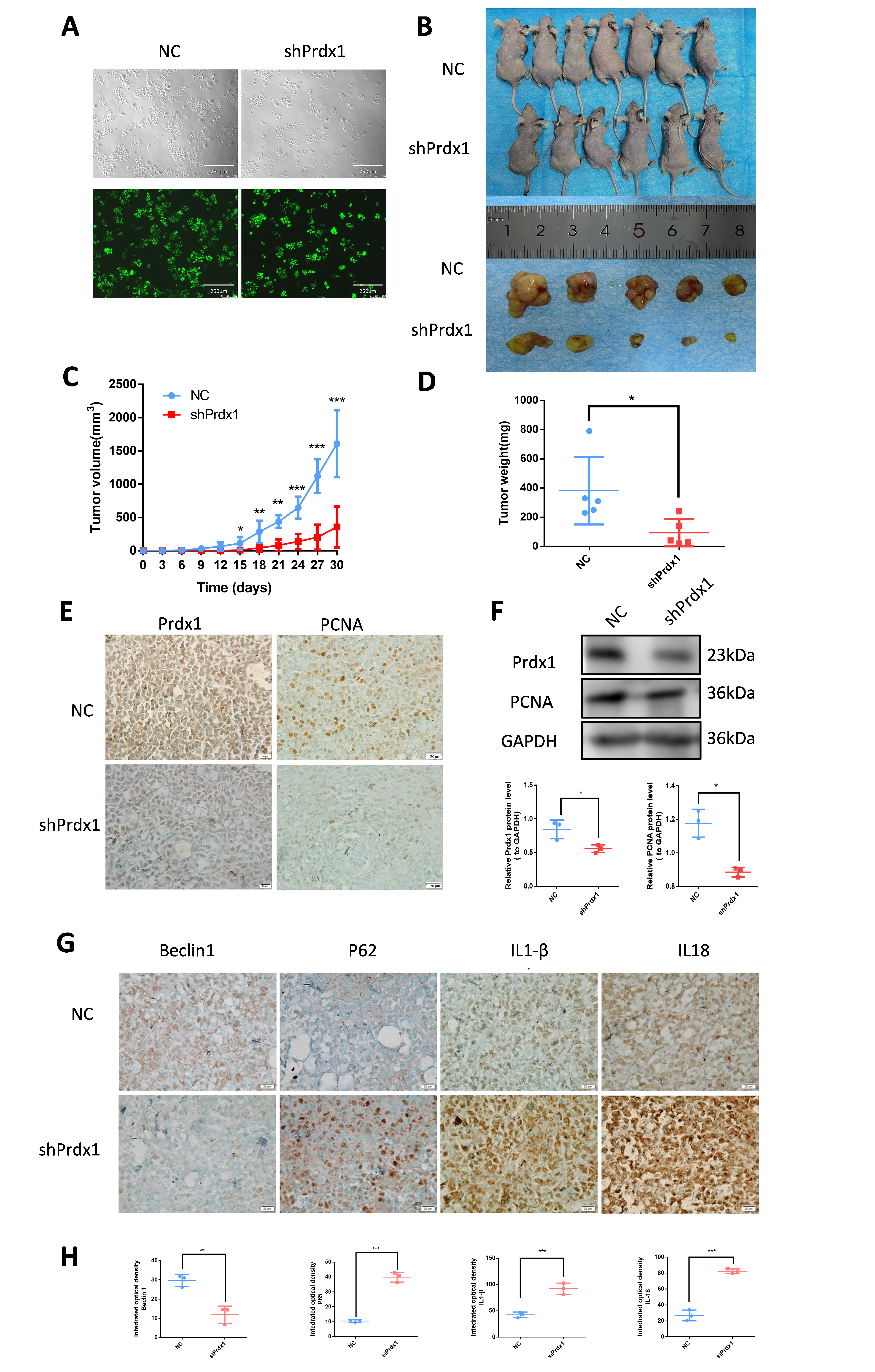

Supplement: Supplementary file 1 — Supplemental Fig. 1 [file 41420_2023_1720_MOESM1_ESM.tif]
