# Supplementary material for: Peroxiredoxin 1 regulates crosstalk between pyroptosis and autophagy in oral squamous cell carcinoma leading to a potential pro-survival
Source: Cell Death Discov. 2023 Nov 25;9:425. doi: 10.1038/s41420-023-01720-7 (PMC10676359; doi:10.1038/s41420-023-01720-7)
Supplement: Supplementary file 2 — Supplemental Fig. 1 legend [file 41420_2023_1720_MOESM2_ESM.doc]

**Supplemental Fig. 1. Prdx1 knockdown inhibits the proliferation of SCC15 cells *in vivo***. A, Quantification of gene transfer efficiency in SCC15 cells was measured by the proportion of fluorescent cells. B, The tumorigenicity of Prdx1 knockdown in SCC15 cells was determined by subcutaneous tumorigenicity test (at day 30) in nude mice (n = 7). C,D, The tumor size (from day 0 to day 30) and tumor weight (at day 30) were compared groups. E, IHC staining of Prdx1 and PCNA in tumor tissue. F, Western blot analysis of Prdx1, PCNA and GAPDH in tumor tissues. G,H, IHC staining of pyroptosis-related proteins and autophagy-related proteins of tumor tissues in NC and siPrdx1 group. The results are shown as the mean ± SD (n=3), ns, non-significant difference；**p*<0.05; ***p*<0.01; ****p*<0.001.
