# Supplementary material for: Peroxiredoxin 1 regulates crosstalk between pyroptosis and autophagy in oral squamous cell carcinoma leading to a potential pro-survival
Source: Cell Death Discov. 2023 Nov 25;9:425. doi: 10.1038/s41420-023-01720-7 (PMC10676359; doi:10.1038/s41420-023-01720-7)
Supplement: Supplementary file 3 — Original Images for Blots and Gels Requirements [file 41420_2023_1720_MOESM3_ESM.pptx]

## Slide 1
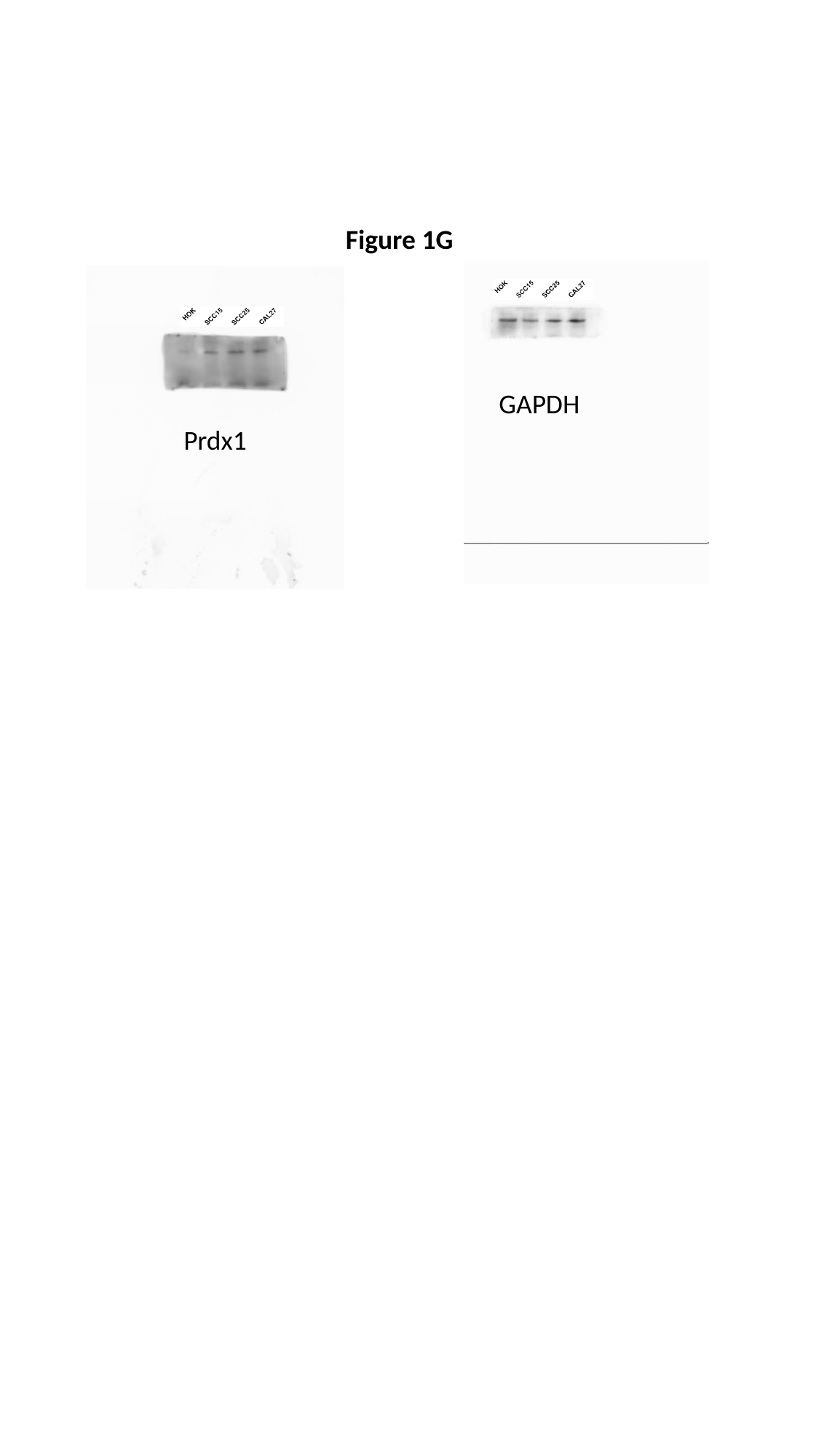

Figure 1G
GAPDH
Prdx1

## Slide 2
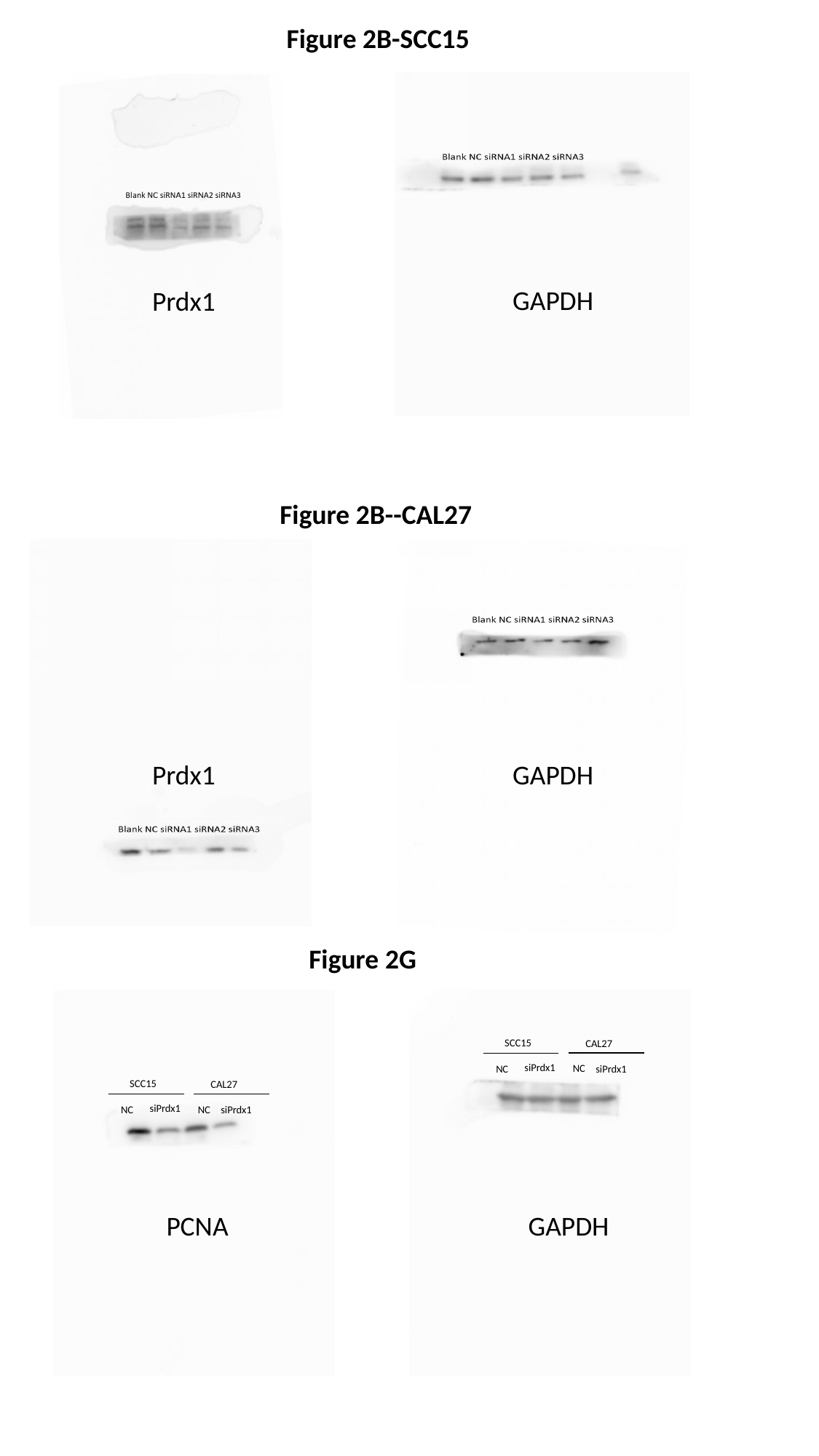

Figure 2B-SCC15
GAPDH
Prdx1
Figure 2B--CAL27
Prdx1
GAPDH
Figure 2G
SCC15
CAL27
siPrdx1
NC
siPrdx1
NC
SCC15
CAL27
siPrdx1
NC
siPrdx1
NC
PCNA
GAPDH

## Slide 3
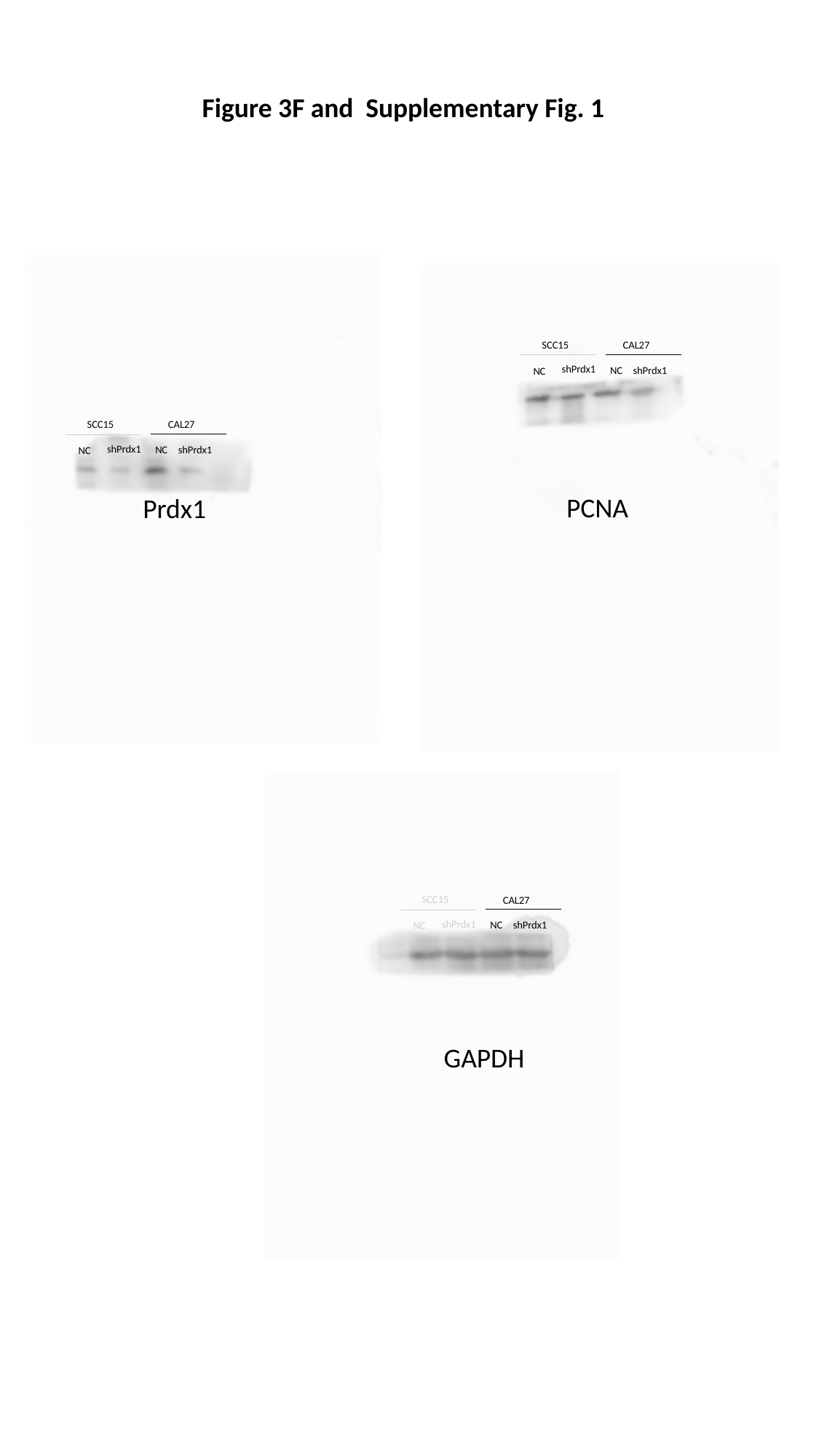

Figure 3F and Supplementary Fig. 1
SCC15
NC
CAL27
NC
shPrdx1
shPrdx1
SCC15
NC
CAL27
NC
shPrdx1
shPrdx1
PCNA
Prdx1
SCC15
NC
CAL27
NC
shPrdx1
shPrdx1
GAPDH

## Slide 4
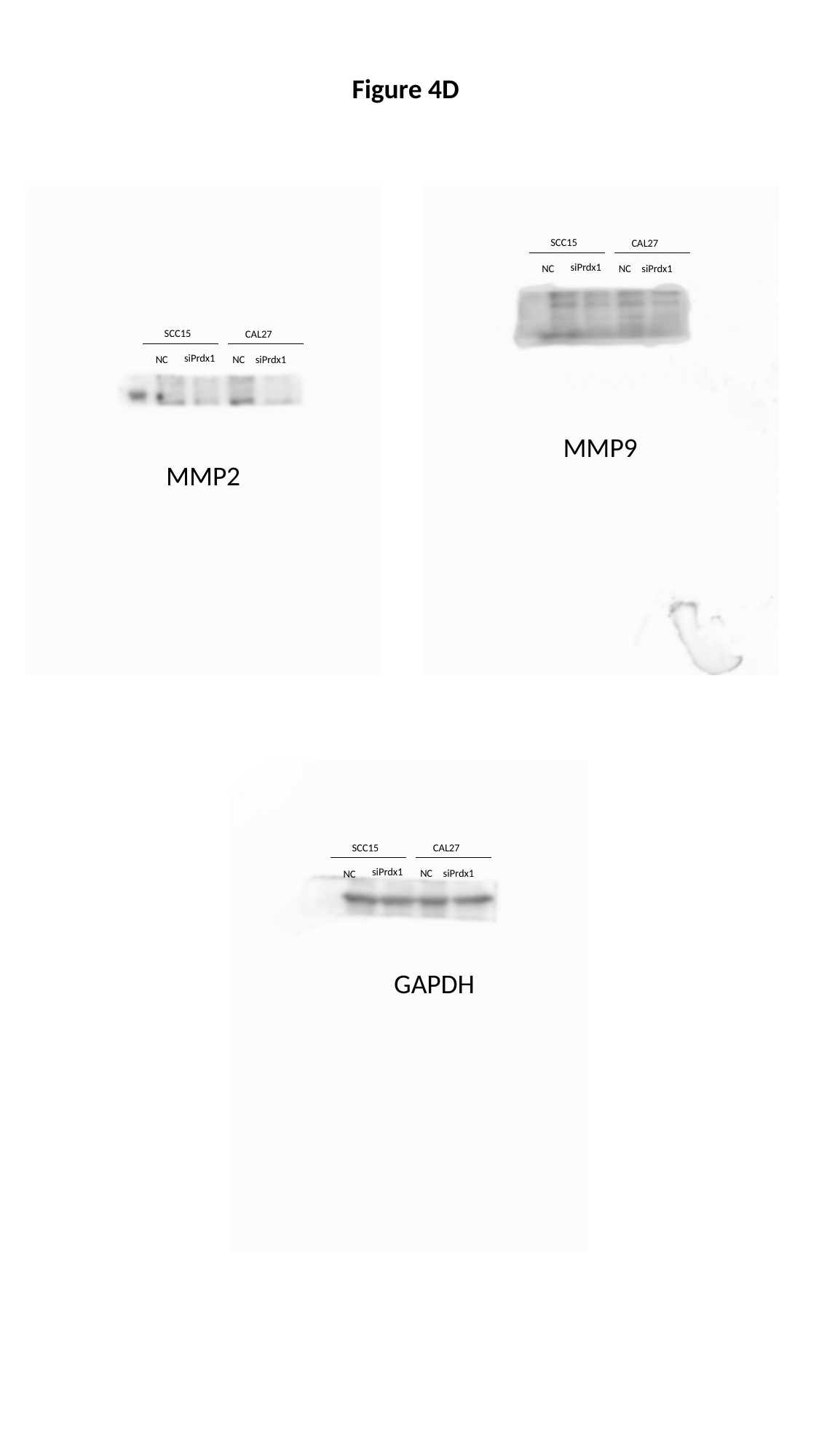

Figure 4D
SCC15
CAL27
siPrdx1
NC
siPrdx1
NC
SCC15
CAL27
siPrdx1
NC
siPrdx1
NC
MMP9
MMP2
SCC15
CAL27
siPrdx1
NC
siPrdx1
NC
GAPDH

## Slide 5
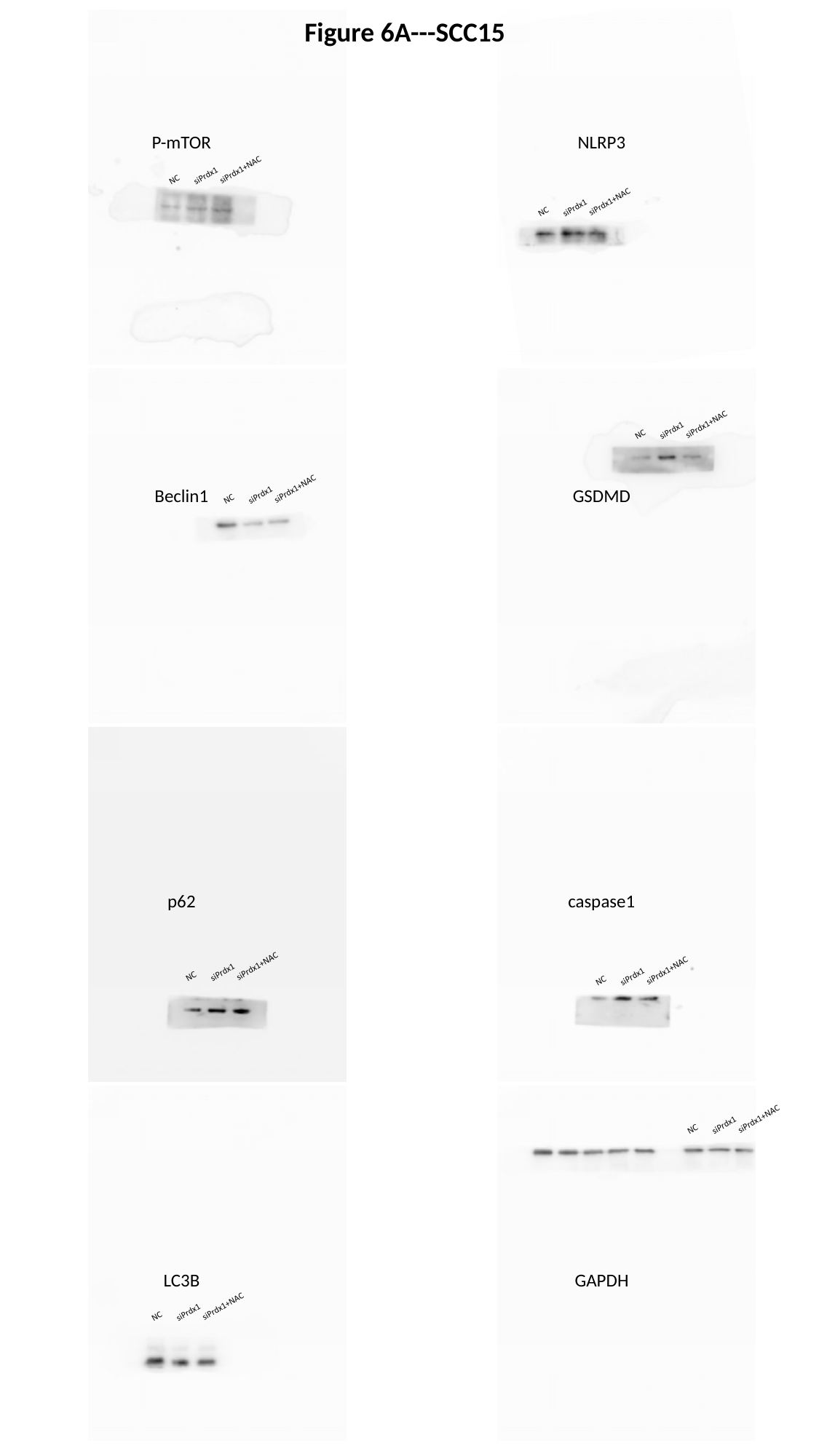

Figure 6A---SCC15
P-mTOR
NLRP3
Beclin1
GSDMD
p62
caspase1
LC3B
GAPDH

## Slide 6
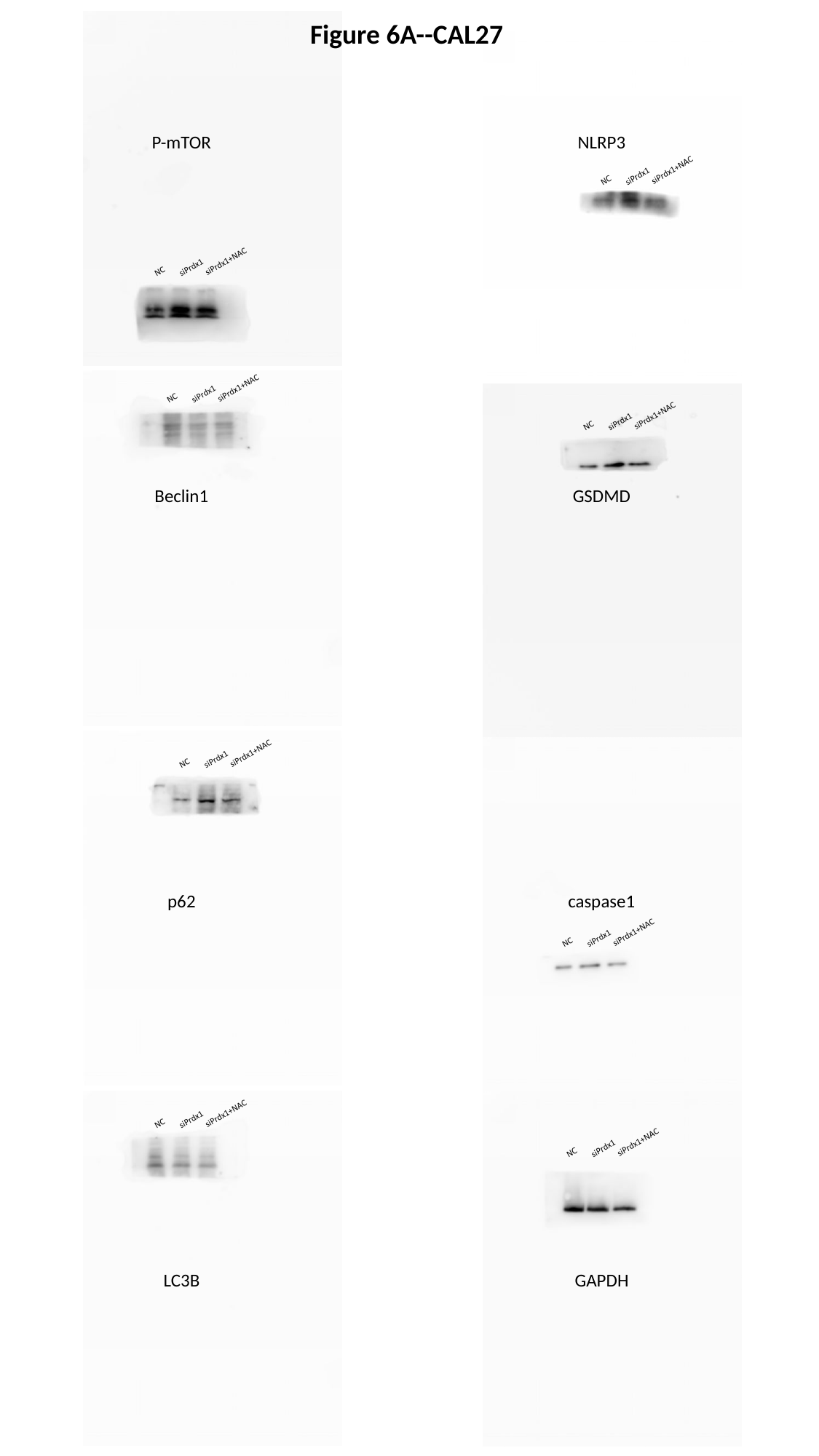

Figure 6A--CAL27
P-mTOR
NLRP3
Beclin1
GSDMD
p62
caspase1
LC3B
GAPDH

## Slide 7
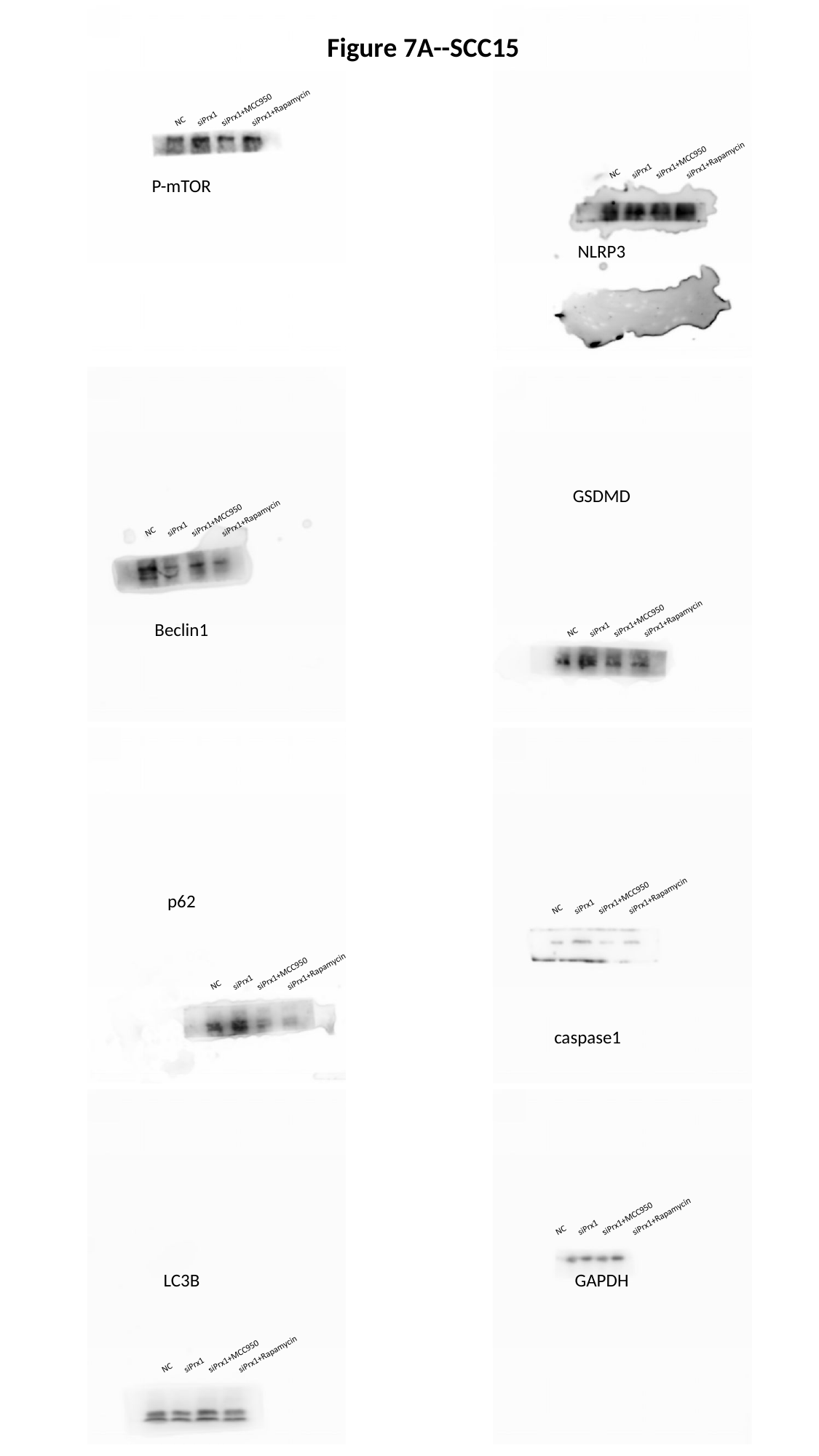

Figure 7A--SCC15
P-mTOR
NLRP3
GSDMD
Beclin1
p62
caspase1
LC3B
GAPDH

## Slide 8
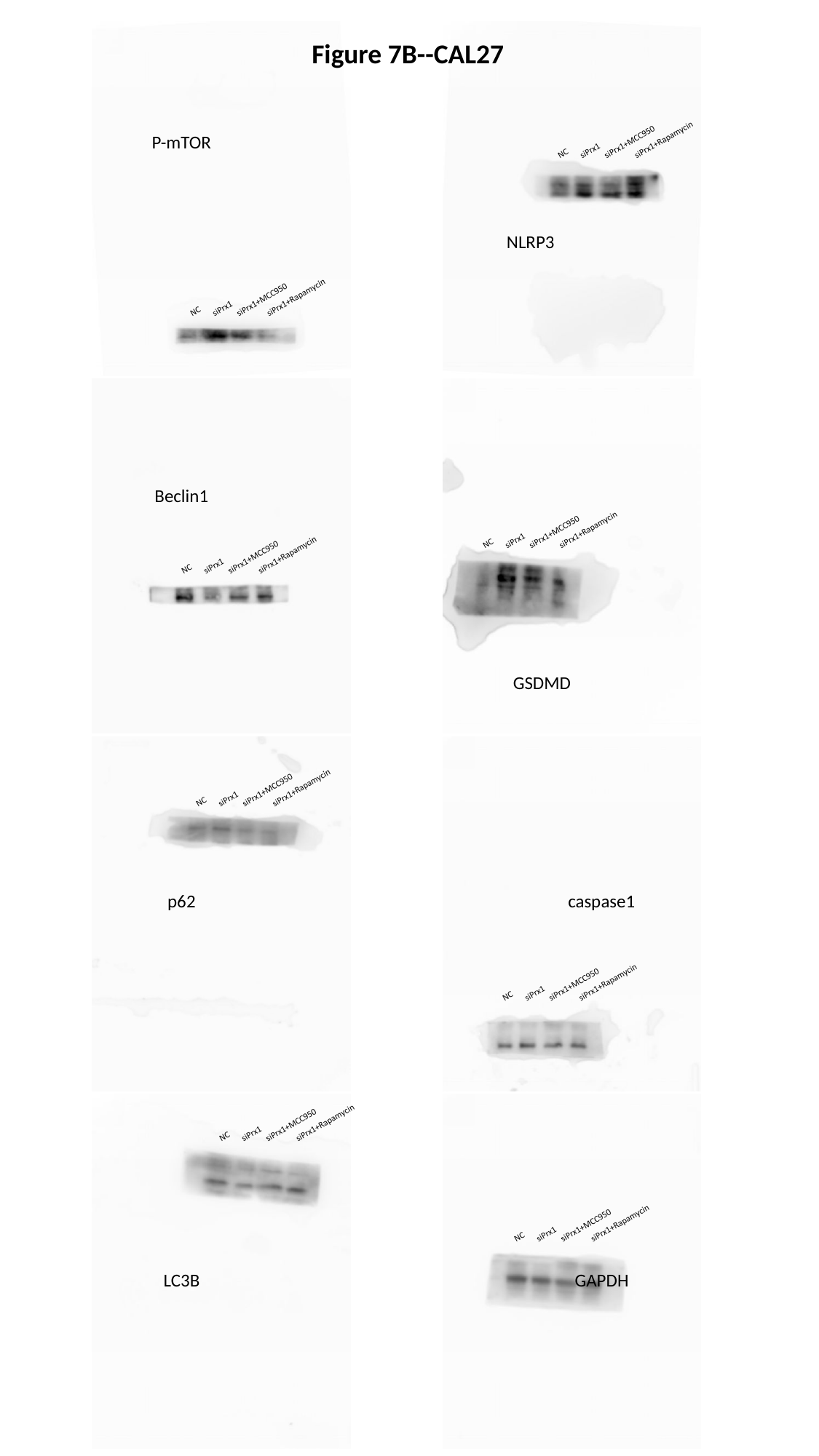

Figure 7B--CAL27
P-mTOR
NLRP3
Beclin1
GSDMD
p62
caspase1
LC3B
GAPDH
